# Varieties of imagery and perception: the structure of task differences

**DOI:** 10.3389/fpsyg.2025.1568148

**Published:** 2025-12-17

**Authors:** Anna Maria Berardi

**Affiliations:** 1 Cognitive Neuroscience Laboratory, Department of Psychology, Harvard University, Cambridge, MA, United States; 2 LCOMS, UFR SHS-Metz, Département de Psychologie, Université de Lorraine, Metz, France

**Keywords:** dorsal vs. ventral streams, visual perception, mental imagery, human, healthy

## Abstract

**Introduction:**

This study assessed the dimensions and factors underlying visual mental imagery abilities in young healthy participants. A second purpose was to compare the underlying pattern of factors and dimensions in imagery with those in the corresponding perception tasks.

**Methods:**

We administered 15 tasks to 32 participants, assessing a wide range of imagery abilities, including imagery for faces, common objects, colors, words, mental rotation, scanning, image maintenance, auditory imagery, and tactile imagery. Response times and error rates were correlated for the imagery and for the perception tasks separately. The matrices were then analyzed using nonmetric multidimensional scaling and principal components analysis.

**Results:**

All analyses indicated the presence of two main clusters, one that appeared to correspond to tasks that draw on the object-properties “ventral system” and one that appeared to correspond to tasks that draw on the spatial-properties “dorsal system.”

**Discussion:**

These results indicate a common segregation of the two major processing systems in visual imagery and visual perception.

## 1 Introduction

Numerous studies have now convincingly demonstrated that two distinct visual processing systems underlie visual perception. These studies provide convergent evidence from effects of lesions in monkey brains (Mishkin et al., 1983), deficits in humans following brain damage (Levine et al., 1985; Farah et al., 1988a,b; Luzzatti et al., 1998), and patterns of brain activation in normal participants (Haxby et al., 1991). In a seminal study, Mishkin et al. (1983) trained monkeys to choose a shape or a food distributor closest to a landmark to obtain a reward. Monkeys with bilateral lesions of the inferior temporal cortex had severe difficulty discriminating shapes (objects), but not in determining their location in space. In contrast, monkeys with bilateral lesions of the parietal lobes had severe difficulty determining the locations of objects, but not in discriminating form. The dorsal system runs from the occipital lobes to the parietal lobes and is involved in encoding spatial properties, such as location and size. The ventral system runs from the occipital lobes to the inferior temporal lobes and is involved in encoding object properties, such as shape and color. These two pathways are also known as the “where” (dorsal) or “what” (ventral) pathways.

In a series of studies of patients with brain lesions, Levine et al. (1985), Newcombe et al. (1987), Farah et al. (1988b), and Farah et al. (1988a) documented dissociable systems of object and spatial representations in visual perception. Patients who have inferior temporal lobe lesions have problems in visual (object) perception that impair their ability to name or recognize visually presented objects, although they can point to them and describe their position appropriately (Farah et al., 1988a). On the other hand, patients who have bilateral parietal damage can name objects presented in their visual fields, but are unable to point to them or describe their location. Farah et al. (1988b) extended these results to visual imagery by describing a patient who was severely impaired in visual (object) imagery, but not in spatial imagery. Researchers have also studied patients who had a dissociation between visual (object) and spatial processing in mental imagery, according to their lesion site, and found that these dissociations parallel those observed in visual perception (Levine et al., 1985; Farah et al., 1988b; Luzzatti et al., 1998).

Nevertheless, researchers have not always observed this overlap in the deficits of perceptual and mental imagery functions, casting some doubt on the roles of the dorsal and ventral visual streams in both visual perception and mental imagery (e.g., Behrmann et al., 1992, 1994; Bartolomeo et al., 1997, 1998; Bartolomeo, 2008; Moro et al., 2008; Committeri et al., 2015; Spagna et al., 2021; Cichy et al., 2012; Pace et al., 2023; Dijkstra et al., 2017). However, other studies provide support for the use of shared pathways in both visual perception and mental imagery (e.g., Kosslyn et al., 1999; Ganis and Schendan, 2011; Senden et al., 2019; Dijkstra et al., 2019; Kosslyn et al., 2001; Pearson et al., 2015).

Brain imaging studies also support the distinction between the dorsal and ventral visual pathways in visual perception (see Ungerleider and Haxby, 1994; Zeki et al., 1991; Haxby et al., 1991, 1993, 1994) and mental imagery (e.g., Kosslyn, 1994; Kosslyn et al., 1998a,b). In addition, Haxby et al. (1991, 1993, 1994) and Ungerleider and Haxby (1994) showed that a face matching task activated, among other areas, the fusiform gyri bilaterally and that a location matching task activated the dorsal occipital, superior parietal, and intraparietal cortex bilaterally. These studies of visual perception, therefore, provided evidence that the processing of spatial characteristics activated the parietal lobes but not the inferotemporal lobes, whereas the processing of faces activated the inferotemporal, but not the parietal, lobes. Similar dissociable activations between object and spatial processing have also been reported in mental imagery (e.g., Kosslyn et al., 1993; Kosslyn, 1994; Kawashima et al., 1995; Ghaëm et al., 1997; D'Esposito et al., 1997; Mellet et al., 1996; Mazard et al., 2004).

Kosslyn (1994) proposed an integrated model based on the idea that visual perception and mental imagery share common processes and brain structures (Kosslyn, 1994; Ganis et al., 2004). Mental imagery, like all complex cognitive functions, is not accomplished by a single process. By “process,” we mean a set of steps that interpret or transform information over time. Mental imagery depends on a number of underlying processes, with sets of processes that often work together being organized into “processing components”. The greater the number of processing components shared by two tasks, the more highly the performance of the tasks should be correlated (Kosslyn et al., 1984, 1990b). The processing components of Kosslyn's model include the visual buffer, the attention window, the spatial properties system, the object properties system, the associative memory, the information shunting, and the attention shifting processing systems (see Kosslyn, 2005). These processing components are themselves divided into a set of more granular and specific processes (interested readers can see Kosslyn et al., 1984, 1990b; Kosslyn, 1994). For example, consider the processes that purportedly are drawn upon when one answers the question: “What shape are a German Shepherd dog's ears?” To answer this question, one has to first access associative memory to retrieve stored information. If one has answered this question previously, it is possible that the verbal classification is stored and can be used to generate a response. However, if the question is novel, one may have to access stored visual memories and create a mental image of the dog. This image is constructed in the visual buffer by accessing stored information about its physical properties, such as color and shape, and its spatial properties, such as the correct relative location of each part or characteristic. The image is reconstructed by the object and spatial processing systems working together. After these systems create the image in the visual buffer, the attention window then needs to focus on the ears of the dog so one can “inspect” their shape, which allows a decision to be made.

The processing components proposed by Kosslyn et al. (1984, 1990b); Kosslyn (1994), which are purportedly shared in visual perception and mental imagery, have been linked to anatomical brain structures. A brain “structure” is an anatomical area that implements specific processing components. For example, the spatial properties processing system has been shown to rely on the dorsal visual pathway, whereas the object properties processing system relies on the ventral visual pathway. These large structures can be decomposed into smaller ones that have more specific functions. For example, area V5 in the dorsal visual pathway has been shown to be involved in the processing of motion for both visual perception and mental imagery, whereas area V4 in the ventral visual pathway has been shown to be involved in processing color in both visual perception and mental imagery (see Zeki, 1990b; Zeki et al., 1991; Simmons et al., 2007). Kosslyn (1994) suggests that visual perception makes use of forward connections from V1 to the frontal lobes, so that when perceiving an object, one can access much more detailed information, whereas mental imagery relies largely on backward connections from the frontal lobes to V1 (see also Van Essen, 1985; Felleman and Van Essen, 1991), where images of objects can be reconstructed from stored information. Previous studies have shown that area V1 has a retinotopic organization in monkeys (e.g., Tootell et al., 1982, 1998; Fox et al., 1987). Kosslyn et al. (1993) further demonstrated a retinotopic organization of the visual cortex (area V1) in humans that is similarly activated in visual perception and mental imagery. For a justification of the brain structures involved in the specific dorsal and ventral IPB tasks reported in this study, see the General Methods and the Introduction sections for each specific task.

The Imagery Processing Battery (IPB) described in this article is designed to assess a wide variety of different aspects of imagery and perception, and it allows us to compare the underlying dorsal and ventral processes in the two domains, given that imagery and perception share at least some common processes (e.g., see Farah, 1985, 1988; Finke, 1989; Kosslyn, 1994; Shepard and Cooper, 1982; Kosslyn et al., 1999; Borst and Kosslyn, 2008; Ganis and Schendan, 2011; Senden et al., 2019; Dijkstra et al., 2019; Kosslyn et al., 2001; Pearson et al., 2015). Rather than fit the data to models, we wanted to examine the structure inherent in the data. We expected the similarities and differences in task performance to reflect the extent to which tasks draw on the same underlying mechanisms. In particular, we expected tasks to cluster based on the segregation of the “dorsal” and “ventral” visual systems. We wanted to use multivariate statistics to discover whether we could find a dorsal/ventral dissociation at a purely behavioral level in both imagery and perception, and whether this dissociation would take the same form in both domains. The knowledge derived from this study would not only extend previous findings in an original manner but also, in a preliminary way, validate the IPB as an appropriate instrument for measuring different types of visual perception and mental imagery abilities relying on the dorsal and ventral streams.

## 2 Methods

### 2.1 Participants

A total of 32 participants between the ages of 18 and 25 were tested; their mean age was 19.3 ± 1.4 (SD) years. All participants but one had at least completed a High School Diploma. Most were current Harvard undergraduates, and a few had earned a college degree from Harvard University. Their mean education was 13.9 ± 1.3 (SD) years. Participants were tested during a 2-h session with a 5-10-min break after half of the tasks were administered. All participants were healthy, as determined by a health questionnaire administered at the beginning of the study, and their vision was normal or corrected to normal (20/25). All participants were right-handed as determined by the Edinburgh Handedness Inventory (Oldfield, 1971). Half the participants were males, and half were females.

### 2.2 General methods

The 15 tasks of the IPB were designed to fall into three major categories: those that rely on the dorsal system, ventral system, or mixed dorsal/ventral systems. We justify our classification based on previous empirical findings, which are cited below. Explanations of the reasons why each task was classified as dorsal, ventral, or mixed are provided in the section where we describe each specific task.

**Figure 1:**
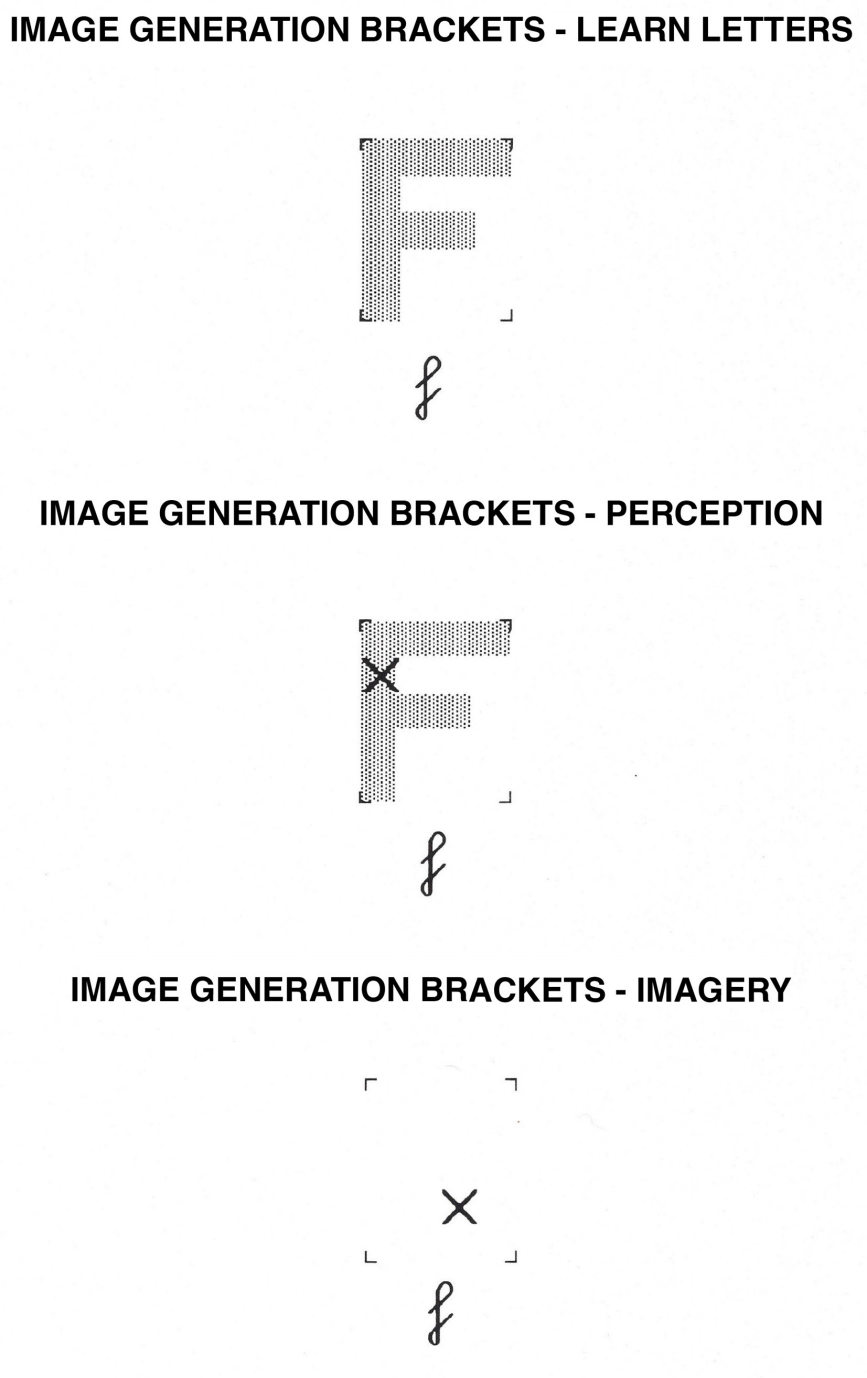
Image Generation Brackets task. In the learning phase, participants learn the shape of uppercase letters within the brackets. In the perception condition, they determine whether or not an “X” falls on the letter while the letter is physically present. In the imagery condition, they determine whether or not an “X” would fall on the letter while they visualize the uppercase version of the letter.

**Figure 2:**
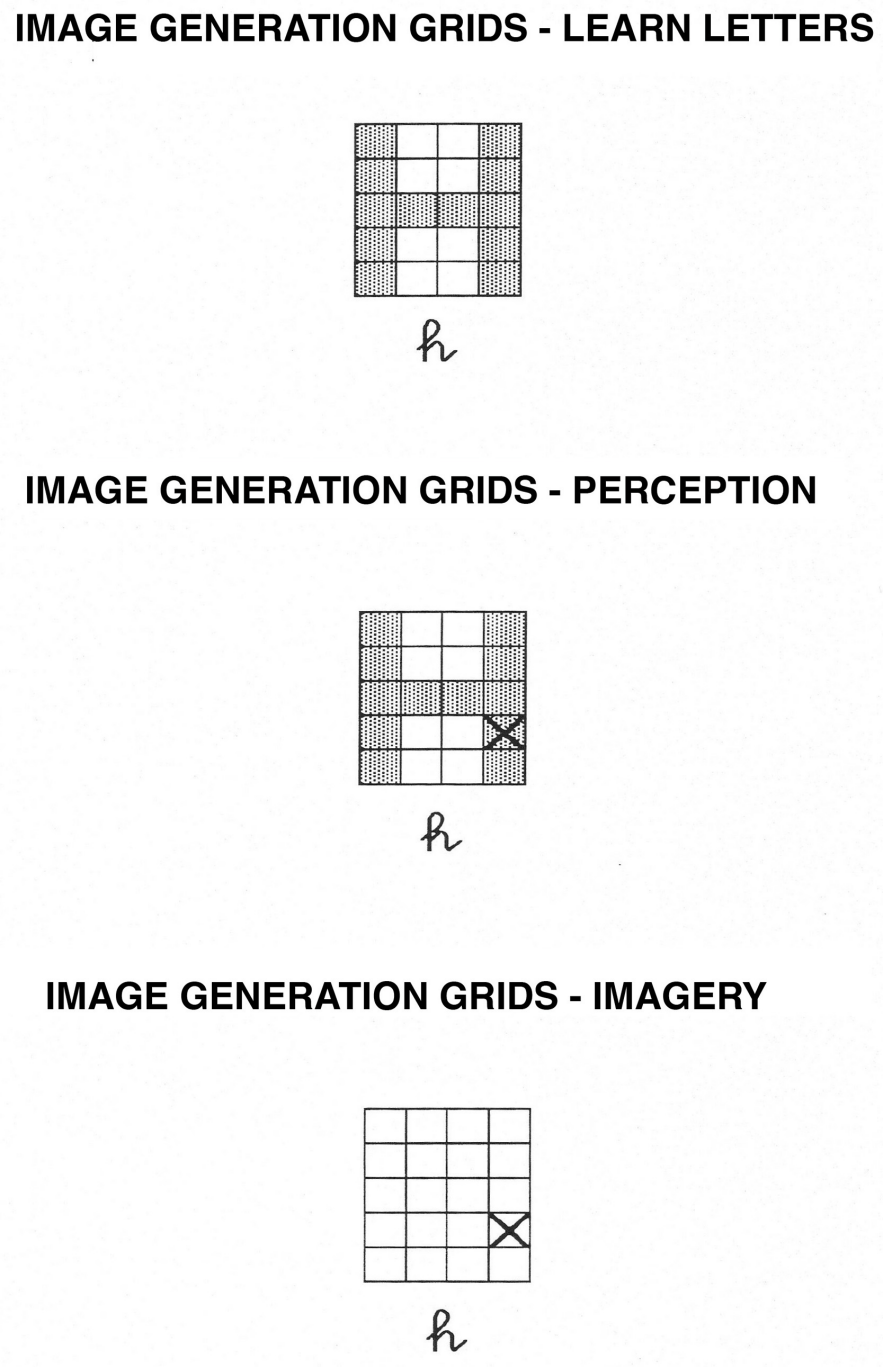
Image Generation Grids task. In the learning phase, participants learn the shape of uppercase letters within the grids. In the perception condition, they determine whether or not an “X” falls on the letter while the letter is physically present. In the imagery condition, they determine whether or not an “X” would fall on the letter while they visualize the uppercase version of the letter.

**Figure 3:**
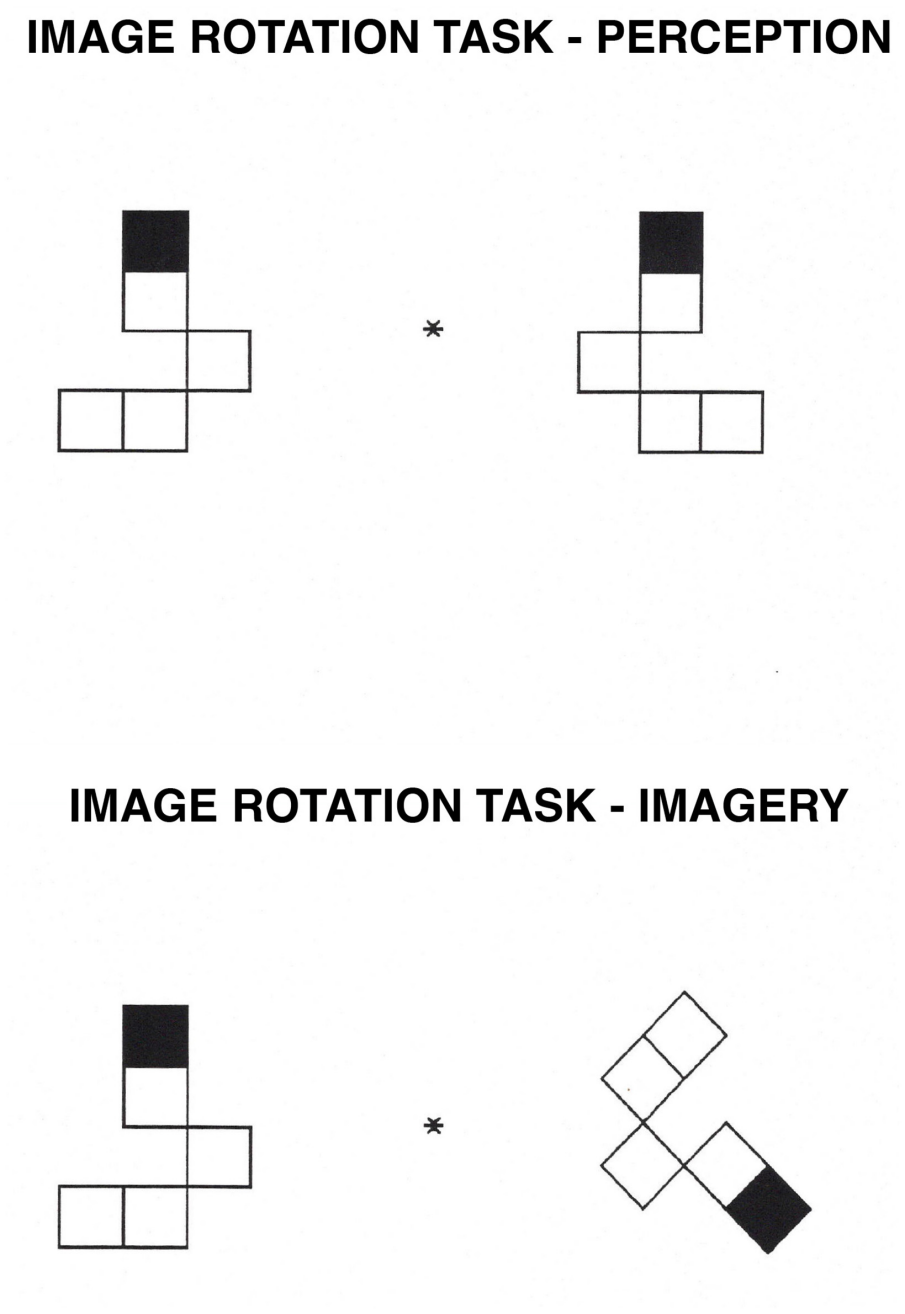
Image Rotation task. In the perception condition, participants decide whether a shape on the right is the same as, or a mirror image of, a shape on the left of a central fixation point. In the imagery condition, participants mentally rotate the image on the right to its upright position and decide whether it is the same as, or a mirror image of, the shape on the left.

**Figure 4:**
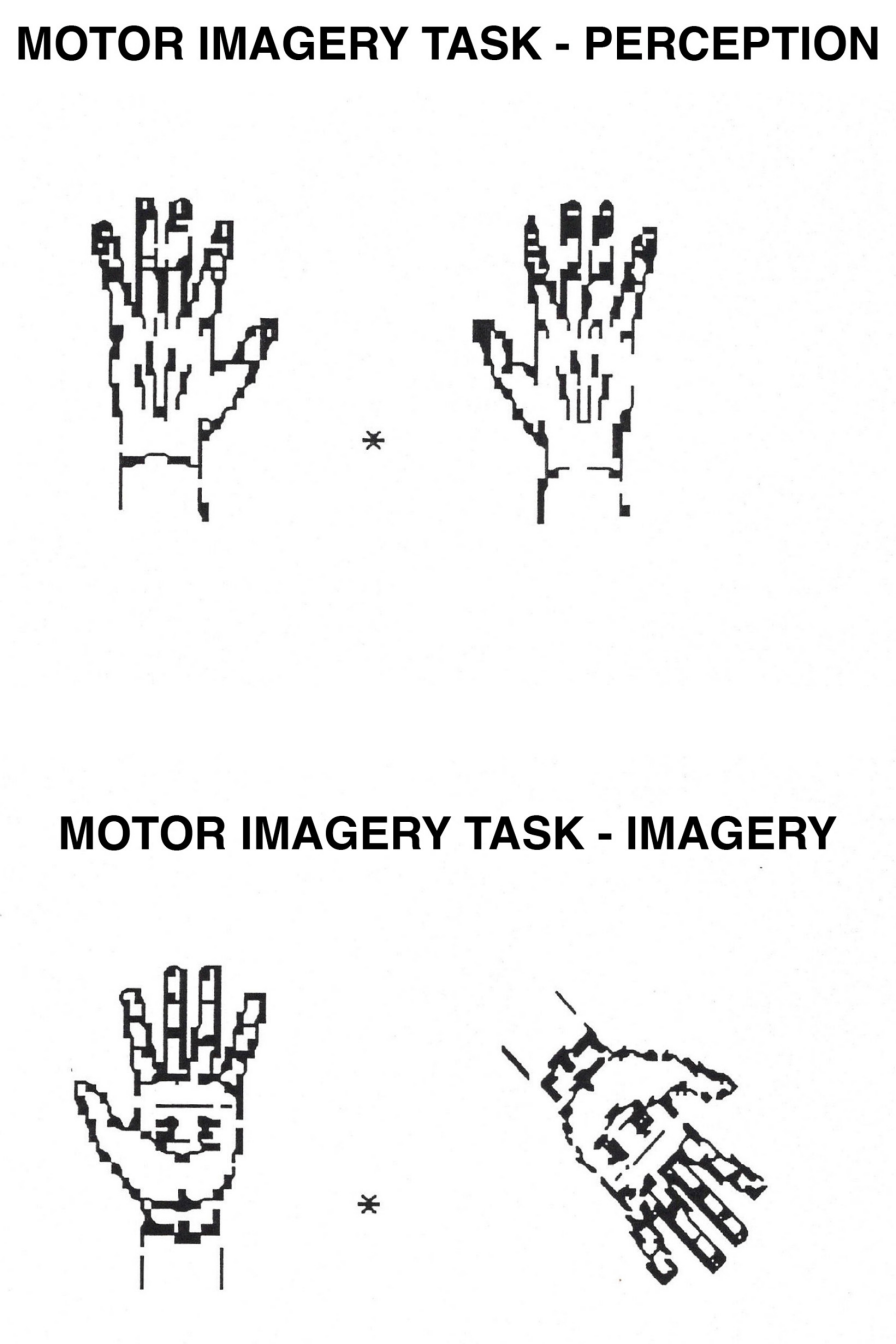
Motor Imagery task. In the perception condition, participants decide whether a hand on the right is the same as a hand on the left of a central fixation point (they are the same if they can be perfectly superposed; they are not if they need to be flipped over first). In the imagery condition, participants mentally rotate the hand on the right to its upright position and decide whether it is the same as the hand on the left (following the same criteria as in perception).

**Figure 5:**
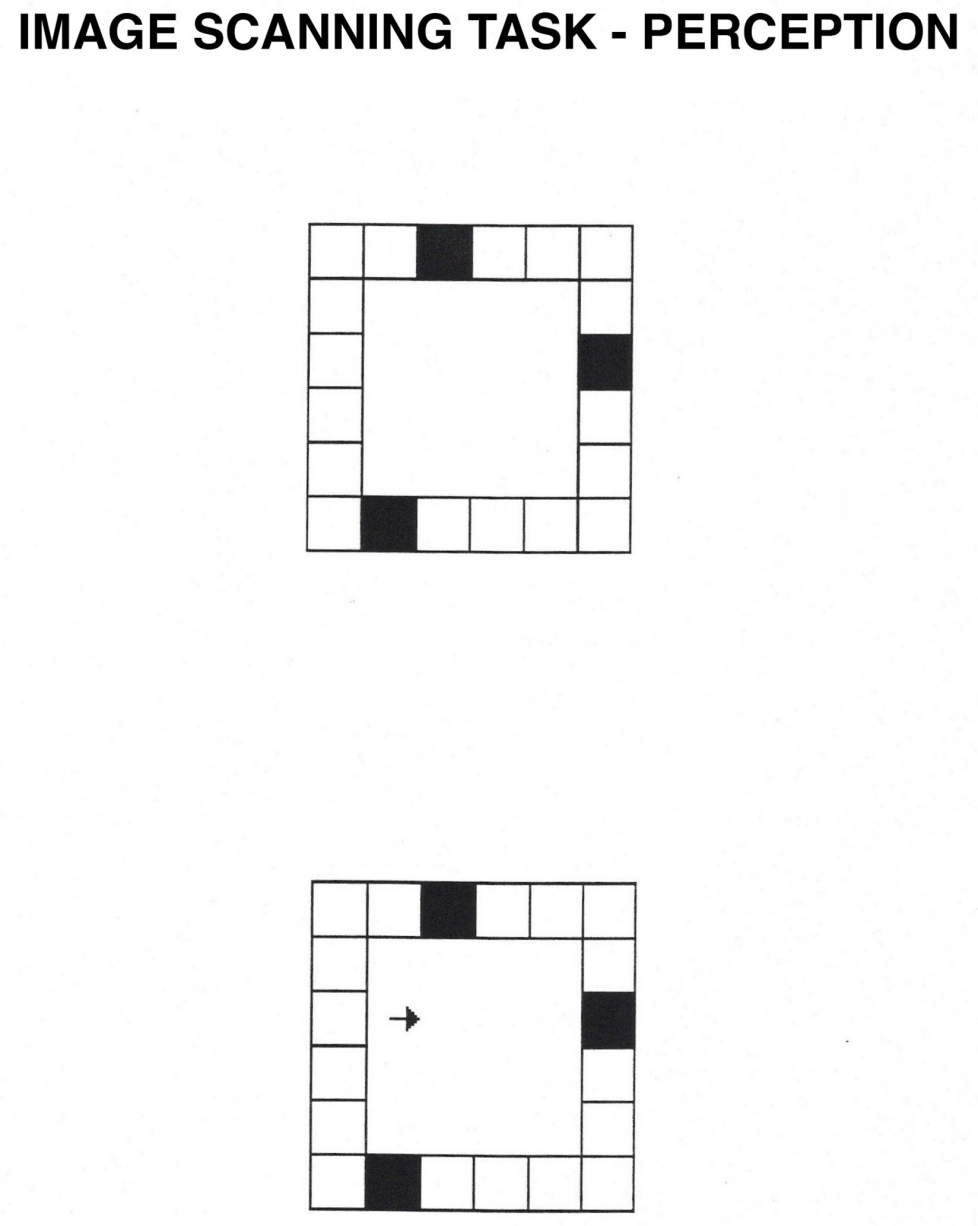
Image Scanning task. In the perception condition, participants see a square grid composed of smaller squares, three of which are black. After they memorize the location of the black squares, they press the spacebar, and an arrow appears. In the perception condition, participants decide whether the arrow points at one of the black squares. In the imagery condition, the arrow flashes for 50 ms inside the display. Then, the entire display disappears, and participants have to decide based on a mental image whether the arrow pointed at one of the black squares.

**Figure 6:**
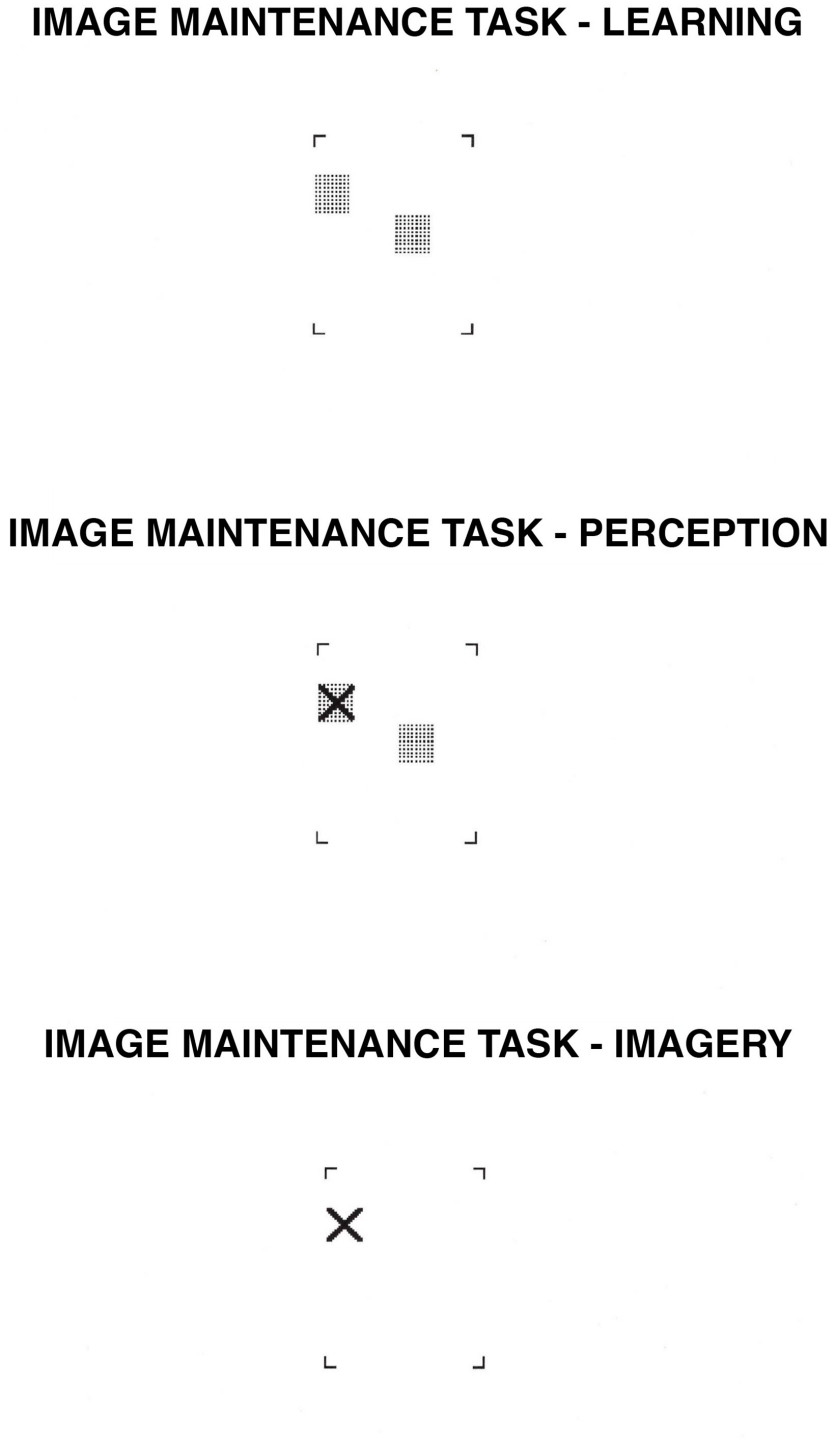
Image Maintenance task. In the learning phase, participants learn the position of either two or four gray squares. After 3 s., an “X” appears somewhere within the brackets. In the perception condition, participants decide whether the “X” falls on one of the gray squares while the squares are physically present. In the imagery condition, participants decide whether the “X” would fall on one of the gray squares based on their own mental images.

**Figure 7:**
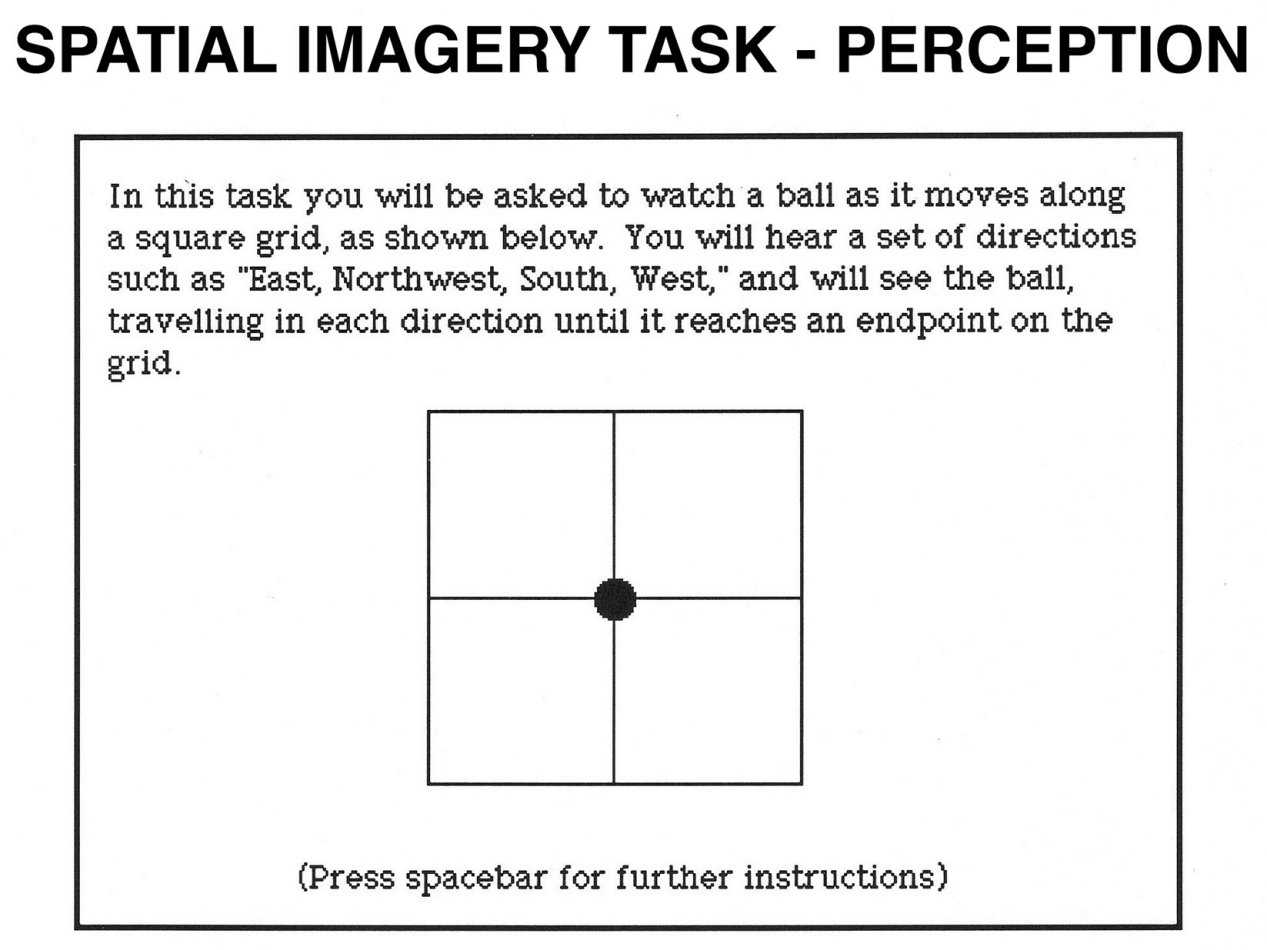
Spatial Imagery task. In the perception condition, participants see a 2 x 2 grid. A filled circle is presented in its center. Each time participants press the spacebar; they hear a direction and see the circle moving in the stated direction. After a varying number of directions, participants hear a cue word (“above,” “below,” “right,” or “left”) and decide whether the word correctly describes the circle's final location relative to its original location. In the imagery condition, participants imagine the circle moving in the stated directions.

The first category included seven tasks that were thought to draw primarily on the spatial-processing, dorsal system. These tasks were image generation within brackets and grids (Kosslyn et al., 1985, 1993; Kosslyn, 1994; Kosslyn et al., 1995, 1998b, 2005; Van der Ham and Borst, 2011; see Figures 1, 2, respectively), image rotation (Crivello et al., 1996; Tagaris et al., 1997; Kosslyn et al., 1998a; Hugdahl et al., 2006; see Figure 3), motor imagery (Kosslyn et al., 1998a; Doganci et al., 2023; Figure 4), image scanning (Finke and Pinker, 1982; Kosslyn et al., 1990a; Dror et al., 1993; Dror and Kosslyn, 1994; see Figure 5), image maintenance (Kosslyn et al., 1990a; Dror and Kosslyn, 1994; see Figure 6), and spatial imagery (Zeki, 1990b; Zeki et al., 1991; Beckers and Homberg, 1992; Zeki et al., 1993; Watson et al., 1993; Sereno et al., 1995; Shipp et al., 1995; Tootell et al., 1995a,b; see Figure 7).

**Figure 8:**
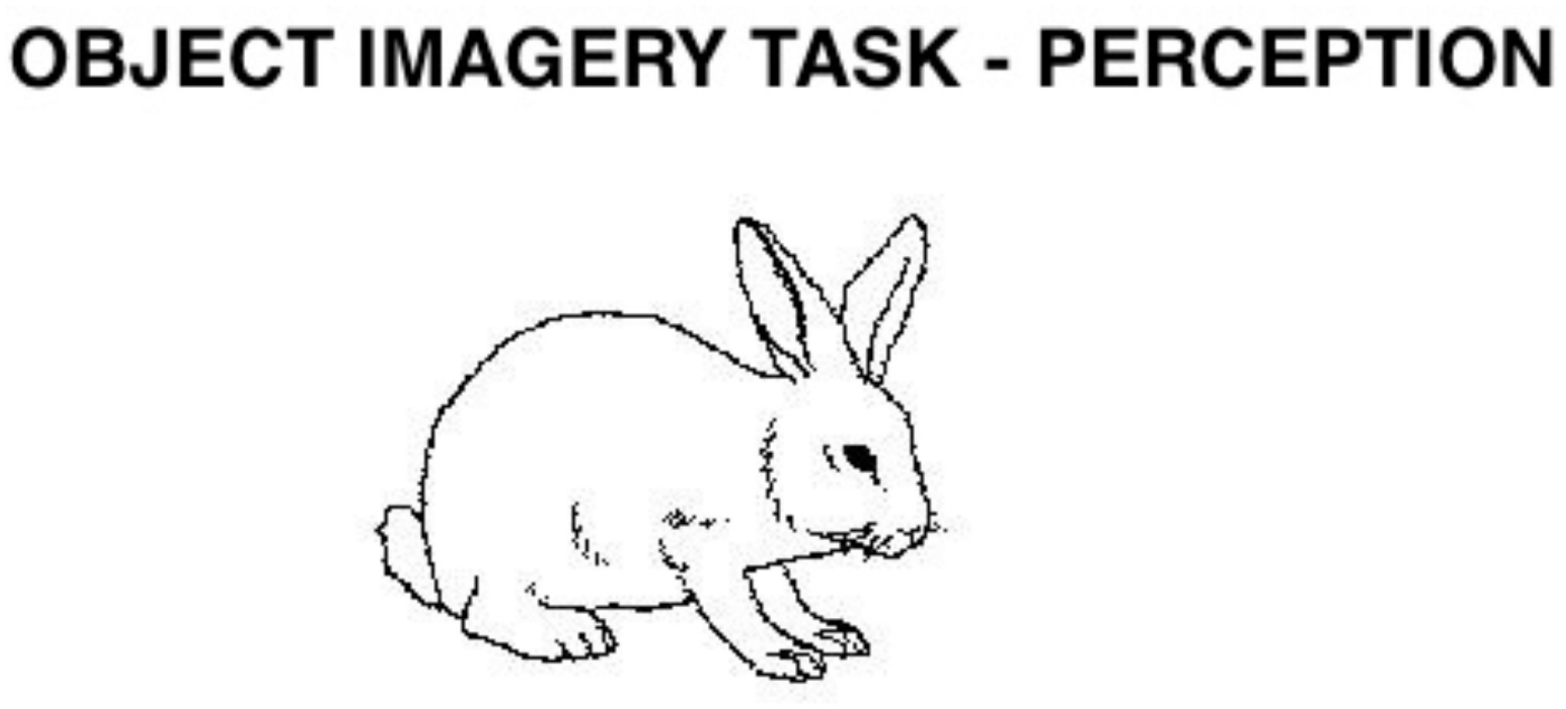
Object Imagery task. Participants hear a sentence describing a feature of an object or animal (such as “A rabbit has long ears”) and decide whether the sentence is true or false. In perception, their decision is based on an actual image presented on the computer screen. In imagery, participants hear the sentence and imagine the corresponding object or animal to decide whether the sentence is true or false. Used with permission of the American Psychological Association, from Snodgrass and Vanderwart (1980), copyright 1980; permission conveyed through Copyright Clearance Center, Inc.

The second category included four tasks that were thought to draw primarily on the object-processing, ventral system. These tasks were object imagery (Goldenberg et al., 1989; Fletcher et al., 1995; Beauregard et al., 1997; D'Esposito et al., 1997; see Figure 8), color imagery (Lueck et al., 1989; Zeki, 1990b; Zeki et al., 1991; Corbetta et al., 1991; Simmons et al., 2007), face imagery (Goldenberg et al., 1989; Haxby et al., 1991, 1994; Andreasen et al., 1996; Clark et al., 1996; Puce et al., 1996; Kanwisher et al., 1997), as well as auditory imagery (Mazziotta et al., 1982; Patterson et al., 2002; Griffiths, 2003; Bendor and Wang, 2005; Alho et al., 2014).

**Figure 9:**
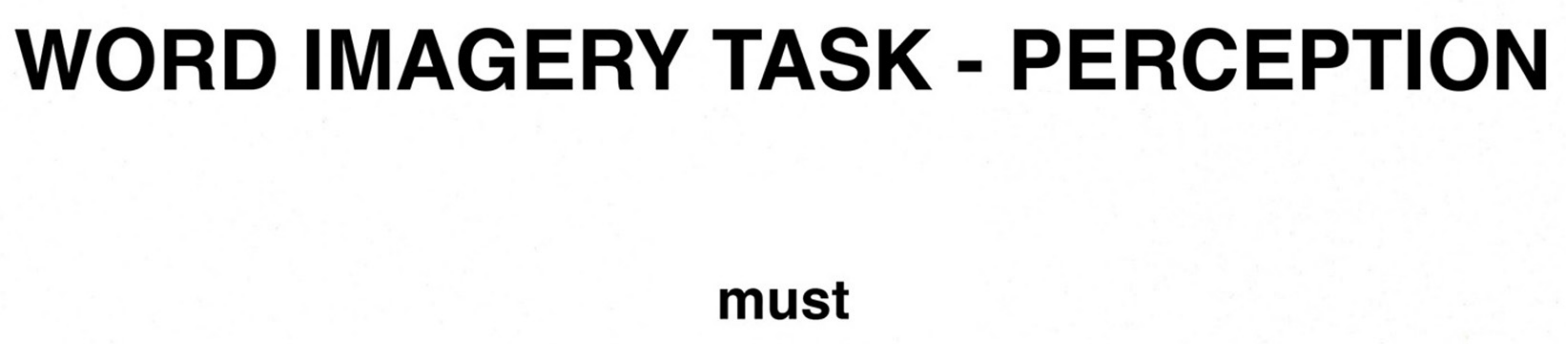
Word Imagery task. In the perception condition, participants see a lowercase word in the middle of the computer screen. In the imagery condition, participants hear a word spoken by the computer and visualize the word printed in lowercase letters. For both tasks, they decide whether or not the first and the last letters of the word are the same height. In the perceptual example given, they are not.

**Figure 10:**
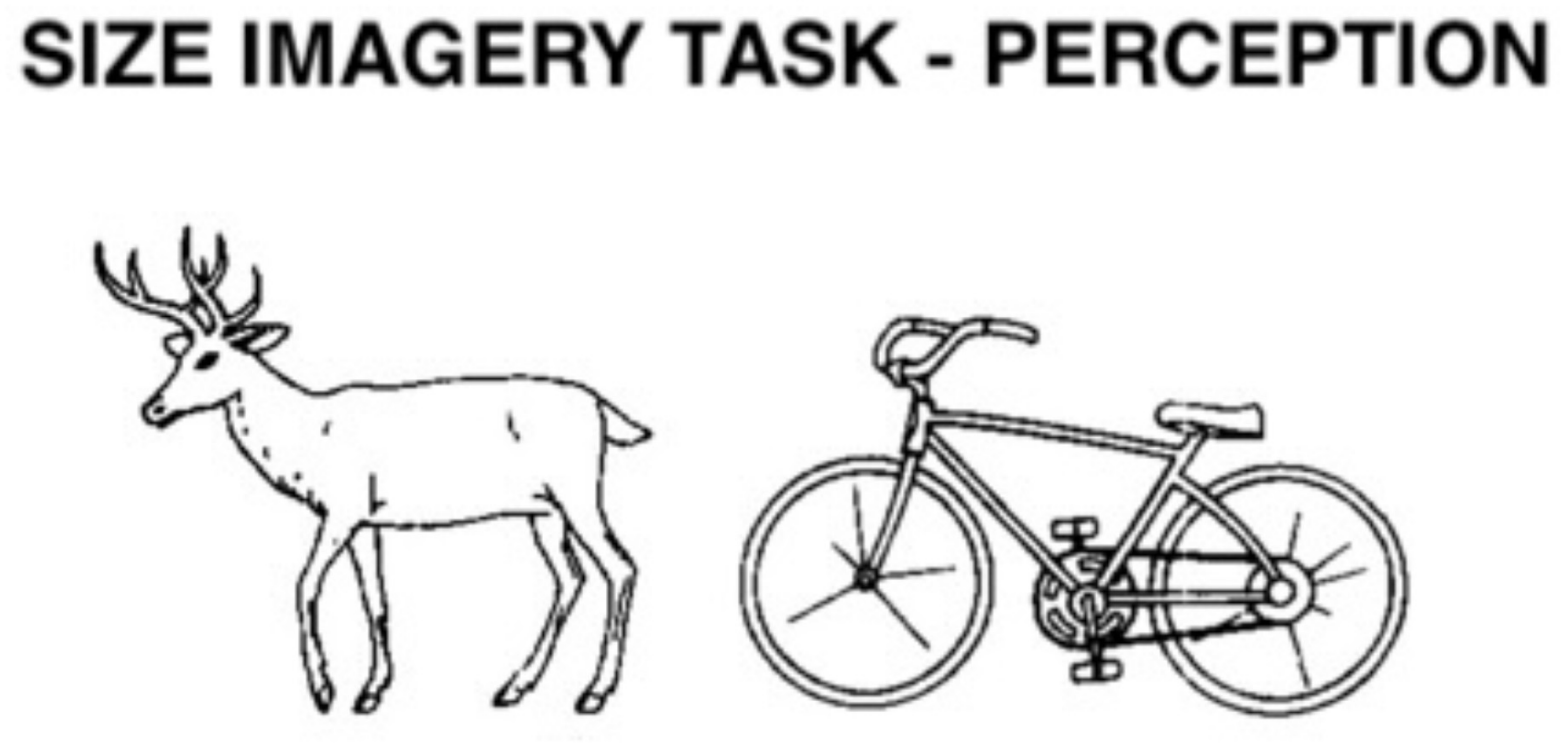
Size Imagery task. In the perception condition, participants see two objects or animals, and at the same time they hear their names (e.g. deer – bicycle). In the imagery condition, they only hear the names of two objects or animals. Participants decide based on the stimuli displayed on the computer screen, or on their own mental images, which of the stimuli is taller. In the example given, the first item is taller. Used with permission of the American Psychological Association, from Snodgrass and Vanderwart (1980), copyright 1980; permission conveyed through Copyright Clearance Center, Inc.

The last category included tasks that were thought to draw on a combination of the two types of processing. These tasks were word imagery (Cohen and Dehaene, 2009; Zhou et al., 2016; see Figure 9), size imagery (Oliver and Thompson-Schill, 2003; see Figure 10), and tactile imagery (Roland and Mortensen, 1987; Uhl et al., 1994; Prater et al., 2004). The distinction among the categories is often subtle, however, because although “objects” are retrieved from long-term memory, the necessary judgment may be “spatial” (such as when comparing the heights of objects). Thus, our assignment of tasks to categories was tentative and based on an analysis of what we considered to be the “rate-limiting” (most difficult) processes in the task; we expected these aspects of the task to determine individual variations that were reflected in our correlation matrices.

#### 2.2.1 Apparatus

All tasks were implemented and administered on a Macintosh PowerBook computer, using the Superlab program (Cedrus Corporation, San Pedro, CA). Audacity and a stereo microphone were used to record sounds. Paintbrush was used to rescale line drawings. Epson Ecotank ET-7700 was used to digitize photographs, and Adobe Photoshop to crop and size them (Adobe Systems Inc., Mountain View, CA); if color balance needed to be adjusted, that was also accomplished with Adobe Photoshop. The screen was adjusted to the maximum level of brightness and contrast. Sounds were produced at a volume of 3.0 (on the Macintosh control panel) through the built-in speaker.

### 2.3 General procedure

Each task had a perception and an imagery condition and included four practice and 16 test trials. The specific questions for each task are presented in the procedure section for that task.

For each of the dorsal visual stream tasks (spatial-properties tasks), the participants generally studied letters or the locations of a set of squares and decided whether an X mark fell on one of those stimuli or an arrow pointed to one of the items at a particular location. In the perception conditions, the stimulus remained visible until a response key was pressed. In contrast, in the imagery condition of each of the spatial-properties tasks, the stimuli were removed before the presentation of the X mark or arrow, and the participants had to visualize the stimulus to make the necessary judgment.

For each of the ventral stream tasks (object-properties tasks), in the perception condition, participants generally saw the picture of an object (or two objects side by side) on the screen and heard a corresponding name of the object(s) in a male voice (reproduced from digitized files on the computer). Participants based their judgments on the picture(s) they saw on the computer screen, and the image disappeared only after they pressed a response key. In the imagery condition of each of these tasks, the participants did not see a display; rather, they heard the name of one (or two) common object(s) and based their judgment on a mental image of the object(s).

For each of the mixed tasks, presented in the same format as the object-properties tasks, participants generally judged the relative size, tallness, or firmness of pairs of objects.

For all tasks relying predominantly on the ventral visual system and for all mixed dorsal/ventral processing tasks, in both perception and imagery conditions, we only used close discriminations. We determined this by having independent groups of participants rate all pairs of stimuli presented in the actual experiments in terms of how subtle the differences were, and we used only those that were rated as being close discriminations and reported as inducing high imagery. For the tactile imagery task, we included close vs. far discriminations. To determine which discriminations to use in the tactile task, we proceeded in the same manner as for all other ventral tasks; an independent group of participants rated using imagery in the close but not in the far discriminations.

We included close discriminations because studies have shown that people use mental imagery when they must discriminate between two similar items from memory (e.g., when they compare two similarly sized objects, such as when they determine which is larger, an orange or a grapefruit), whereas people do not use imagery to discriminate between two dissimilar items (“far discriminations”, e.g., deciding which is larger, an elephant or a mouse). For a detailed justification and empirical evidence, see Chapter 9 of Kosslyn (1980), Kosslyn et al. (1977), and Farah et al. (1988b). Similarly, Eddy and Glass (1981) asked participants to rate whether they used mental imagery in close vs. far discriminations and found evidence that mental imagery was indeed used for close, but not for far discriminations. The distinction between close vs. far discriminations can be applied to different object properties (such as differences in size, color, or the roughness of two objects). In fact, our participants did report that they used imagery when making close discriminations for the items used in the IPB tasks. To select these items, we first asked groups of participants the degree to which they used imagery to make each comparison, and we included only comparisons for which they reported using imagery.

Participants sat in a quiet room at a distance of 50 cm from the computer monitor. Instructions were always presented on the computer screen and read by the participants. For the ventral visual tasks, there were two alternate and equivalent test versions; we designed two versions to prevent the participants from remembering the items or their judgments in the perception condition and using them in the imagery condition, or vice versa. The comparability of the two tasks was based on ratings of similarity obtained by an independent group of 10-16 participants, as will be described in the appropriate test sections. When the tasks had two versions, half the participants got Version 1 in the perception condition and Version 2 in the imagery condition, and the other half had the reverse assignment. The order of administration of the tasks (1-15 or 15-1) and the order of the conditions (perception and imagery) were counterbalanced across participants.

Tasks tapping the dorsal and/or ventral systems were intermixed. We administered the tasks in the following order for half the participants, and in the reverse order (except that the response training task was always first) for the other half: response training task, object imagery, image generation (brackets), word imagery, image rotation, tactile imagery, image scanning, size imagery, image generation (grids), auditory imagery, color imagery, image maintenance, spatial imagery, face imagery, and motor imagery. After the last task was administered, the response training task was re-administered to determine whether there were any changes in response times either due to practice or fatigue effects. All tasks and each condition (perception or imagery) had half “Yes” and half “No” correct responses. The trials were arranged randomly but with the constraint that no more than three “Yes” or three “No” responses could occur in succession. The “Yes” key was the letter “b” on the computer keyboard, and the “No” key was the letter “n”; the keys were labeled “Y” for “Yes” and “N” for “No”. All participants responded with their right hand and pressed the “Y” and “N” keys with their index and middle fingers, respectively. Participants advanced to the next stimulus by pressing the spacebar with the thumb of their dominant hand. Participants were instructed to respond as quickly and accurately as possible.

Items in all tasks were “mini-blocked,” so that each combination of variables being manipulated appeared once before any appeared two times, and then all appeared two times before any could appear three times, and so on. This design controls for the selective effects of practice or fatigue on specific variables within the task. During the practice trials, the computer beeped when the incorrect response key was pressed, but no such feedback was provided during the test trials. Response accuracy and response times were the dependent measures for all tasks.

### 2.4 Statistical procedures

Mean accuracy and mean response times were computed for all tasks in the battery and for the perception and imagery conditions separately. We excluded response times when participants made an error and excluded response times greater than two standard deviations above each participant's mean within a cell. Response accuracy and response times were analyzed using analyses of variance with either one (condition) or two (condition, complexity) within-participant factors, to determine whether the tasks showed the expected imagery and complexity effects, which would indicate that the measures reflect the type of processing of interest.

Mean error rates and response times (±SD) are shown for perceptual and imagery conditions separately in Table 1.

**Table 1:** Mean error rates (%) and response times (ms) for IPB tasks.

		**Perception (mean** ±**SD)**		**Imagery (mean** ±**SD)**	
		**ER**	**RT**	**ER**	**RT**
**Response training**					
Session 1		3.13 ±5.72	440 ±66	——–	——–
Session 2		0.78 ±2.10	441 ±43	——–	——–
**Dorsal system tasks**					
Image generation brackets	Easy	1.56 ± 4.20	676 ± 158	1.17 ± 3.70	1,082 ± 239
	Difficult	2.73 ± 6.91	692 ± 219	8.59 ± 11.64	1,377 ± 461
	Near	0.78 ± 3.07	670 ± 157	2.34 ± 5.89	1,118 ± 255
	Far	3.52 ± 7.26	700 ± 218	7.42 ± 8.90	1,312 ± 413
Image generation grids	Easy	0.78 ± 3.07	689 ± 92	2.34 ± 4.96	1,191 ± 300
	Difficult	1.17 ± 3.70	735 ± 104	3.13 ± 7.10	1,436 ± 495
	Near	0.78 ± 3.07	706 ± 104	1.56 ± 4.20	1,123 ± 258
	Far	3.52 ± 7.26	714 ± 86	3.91 ± 6.69	1,305 ± 413
Image rotation	0°	2.23 ± 3.81	1,046 ± 215	——–	————
	90°	——–	——–	9.77 ± 15.14	2,439 ± 677
	135°	——–	——–	8.98 ± 10.16	2,628 ± 825
Motor Imagery	0°	2.34 ± 4.42	873 ± 194	——–	——–
	90°	——–	——–	2.34 ± 4.96	1,372 ± 325
	135°	——–	——–	2.73 ± 6.91	1,604 ± 501
Image scanning	Near	1.56 ± 4.20	767 ± 135	5.08 ± 10.93	690 ± 85
	Far	1.56 ± 4.20	826 ± 118	5.86 ± 11.87	738 ± 100
Image Maintenance	2 squares	1.56 ± 4.20	717 ± 196	4.30 ± 6.82	1,120 ± 290
	4 squares	1.56 ± 5.27	723 ± 201	5.86 ± 10.03	1,323 ± 483
Spatial imagery	Short	4.69 ± 7.61	1,229 ± 230	8.20 ± 9.84	1,378 ± 442
	long	3.13 ± 6.35	1,150 ± 241	8.20 ± 11.27	1,259 ± 350
**Ventral system tasks**					
Object imagery		6.06 ± 7.01	2,669 ± 217	8.59 ± 8.36	2,874 ± 306
Face imagery		1,055 ± 9.97	1,315 ± 512	23.05 ± 13.51	2,022 ± 525
Color imagery		9.18 ± 7.44	2,191 ± 388	25.78 ± 10.50	2,572 ± 436
Auditory imagery		13.09 ± 7.67	2,121 ± 625	23.63 ± 9.22	2,693 ± 947
**Mixed processing tasks**					
Word imagery		4.30 ± 4.88	941 ± 157	6.25 ± 8.55	1,758 ± 276
Size imagery		4.69 ± 9.91	1,028 ± 327	24.22 ± 12.48	2,322 ± 819
Tactile imagery		6.82 ± 15.91	1,583 ± 311	41.25 ± 13.26	2,126 ± 536

We correlated response times in the imagery tasks using Pearson correlations, and the obtained correlation matrix was analyzed with nonmetric multidimensional scaling (MDS) based on monotonicity coefficients (Shepard, 1962; Kruscal, 1964) with a maximum of 50 iterations to determine the distribution in space of 14 of the 15 IPB tasks; we did not include the response training task because it was not an imagery task. We conducted the same analysis with the response times from the perceptual analogs of the imagery tasks. We then analyzed the response times from the 14 IPB tasks with factor analysis (Harman, 1976), to confirm the interpretation of the visual patterns obtained with MDS. The same MDS and factor analyses were also carried out on the error rates for both perceptual and imagery conditions. All statistical analyses were carried out with SPSS (Armonk, NY: IBM Corp).

### 2.5 Tasks

#### 2.5.1 Response training

The primary purpose of this task was to familiarize participants with the general procedure we used for all tasks in the IPB. The response training task was administered two times: once at the beginning and once at the end of the testing session.

##### 2.5.1.1 Method

###### 2.5.1.1.1 Materials

The word “YES” or “NO” was presented in uppercase letters at the center of the computer screen. Words subtended a visual angle of 2.3° horizontally and 1.2° vertically. A total of 16 words were presented one at a time in black on a white background. Half were “Yes” and half were “No”.

###### 2.5.1.1.2 Procedure

An exclamation point was presented at the center of the screen; participants focused on it and pressed the spacebar to see the next stimulus. After 500 ms, the word “YES” or “NO” appeared and participants pressed the “Y” or “N” key, as appropriate. Words remained on the computer screen until the participant pressed a response key. After that, the exclamation point returned.

##### 2.5.1.2 Results and discussion

###### 2.5.1.2.1 Error rates

Participants made more errors on the first administration of this task than on the second (3% vs. 1%), *F*_(1, 31)_ = 4.73, *p* = 0.04.

###### 2.5.1.2.2 Response times

Participants had equivalent response times on the first and second administrations (440 vs. 441 ms), *F* < 1.

In short, we had evidence for effects of practice, not of fatigue; such effects did not alter response times but did reduce error rates; this result may simply indicate that the participants were more at ease with the task on the second administration, perhaps because they were over practiced with pressing buttons after all the tasks had been administered.

#### 2.5.2 Image generation: brackets

We designed this and the following six tasks to rely predominantly on processing in the dorsal system. Image generation is the process through which visual images are formed by activating stored visual information. This task was originally designed by Podgorny and Shepard (1978), and several variations were created by Kosslyn and his collaborators (e.g., Kosslyn et al., 1988, 1993, 1995; Dror et al., 1993; Dror and Kosslyn, 1994). In this task, participants decide whether an X covers an uppercase letter while the letter is present or while participants visualize it. Several effects have now been consistently reproduced in the imagery condition of this task. Participants take more time and make more errors when deciding whether an X covers a more complex letter than a simpler one (i.e., one with fewer segments). In addition, they take more time and make more errors when the X is placed on a segment that typically is drawn later in the sequence of strokes than when it is placed on a segment that is drawn early in the sequence. In the perceptual versions of this task, neither response times nor error rates are affected by these variables (Kosslyn et al., 1988).

This task was classified as spatial because the segments of the letters need to be positioned appropriately and also because previous positron emission tomography experiments have indicated that this version of the task activates the parietal but not the temporal lobes (Kosslyn et al., 1985, 1993, 2005; Kosslyn, 1994).

##### 2.5.2.1 Method

###### 2.5.2.1.1 Materials

Uppercase letters in shaded gray were presented at the center of the computer screen within four brackets, which formed the corners of an imaginary 4 × 5 grid. The letters F, G, C, and P were used as stimuli; two letters had 3 segments (F, C), and two letters had 4 and 5 segments, respectively (P, G). Letters subtended a visual angle of 3° horizontally and 3.6° vertically. On half the trials for each letter, the X would fall on the letter and on the other half, it would not. On half the trials, the X appeared on an “early” segment (i.e., one typically drawn early in the sequence, as determined by Kosslyn et al., 1988) or adjacent to it, and on the other half it would fall on a “late” segment or adjacent to it. For an example of the stimuli used, see Figure 1.

###### 2.5.2.1.2 Procedure

This task began with a learning phase in which participants familiarized themselves with the letters used in the experiment proper. Participants saw an exclamation point at the center of the computer screen, and 400 ms after pressing the spacebar, a lowercase letter was presented centrally below the four brackets. The uppercase version of the same letter was presented simultaneously within the brackets. Participants were instructed to study the uppercase version of each letter and remember exactly how it looked. The participant then pressed the spacebar, and 400 ms later, another letter was presented. After all letters were presented three times each in a random order (with the constraint that all letters had to be presented once before any letter would be repeated, two times before any was presented three times, and so on), participants drew the letters from memory within empty sets of brackets on a piece of paper. When participants pressed the spacebar, the lowercase version of a letter appeared at the center of the screen. Participants drew the corresponding uppercase version of the letter on the paper as accurately as possible without time constraints. When participants finished drawing one letter, they pressed the spacebar, and another letter was presented. If all uppercase letters were drawn correctly, the participant would now proceed with the perceptual or imagery version of the task. All participants were able to draw the letters correctly on the first attempt.

In the perception condition, the test trials had the following form: an exclamation point appeared until the participant pressed the spacebar to indicate that he or she was ready to start the next trial. After 500 ms, one of the lowercase letters studied appeared for 500 ms at the center of the screen. After 500 ms, it was replaced by that lowercase letter presented below a set of 4 brackets. At the same time, the uppercase version of the letter appeared within the brackets, with an X at a particular location within the brackets. The participants decided whether the X fell on the uppercase version of the letter. If the X fell on the letter, participants pressed the “Y” key; if it did not, they pressed the “N” key. After a response key was pressed, the exclamation point returned to the screen until participants pressed the spacebar to start the next trial.

In the imagery condition, the procedure was the same in all respects but one: When the set of four brackets appeared, the uppercase version of the letter was not present. Participants visualized how the uppercase version of the letter appeared within the brackets and then decided whether or not the X fell on the letter.

##### 2.5.2.2 Results and discussion

###### 2.5.2.2.1 Error rates

Participants made more errors in the imagery condition than in the perception condition (5% vs. 2%), *F*_(1, 31)_ = 5.96, *p* = 0.02. Although in the perception condition, participants made an equivalent amount of errors with the simple and complex letters (2% vs. 3%), *F* < 1, in the imagery condition participants made more errors with the more complex letters (1% vs. 9%), F_(1, 31)_ = 11.77, *p* = 0.002. In the perception condition, there was a trend for participants to make more errors with late than early probes (4% vs. 1%), F_(1, 31)_ = 3.52, *p* = 0.07, and this difference was significant in the imagery condition (7% vs. 2%), F_(1, 31)_ = 10.42, *p* = 0.003.

###### 2.5.2.2.2 Response times

Participants were slower in the imagery condition than in the perception condition (1230 vs. 684 ms), *F*_(1, 31)_ = 140.24, *p* = 0.0001. In the perception condition, response times for the simple and complex letters were comparable (676 vs. 692 ms), F < 1. In the imagery condition, participants were slower with the complex letters (1,377 vs. 1,082 ms), *F*_(1, 31)_ = 26.65, *p* = 0.0001. When probe distance was analyzed, in the perception condition there was a trend for slower responses with the late than the early probes (700 vs. 670 ms), *F*_(1, 31)_ = 3.06, *p* = 0.09, but this trend was significant in the imagery condition (1,312 vs. 1,118 ms), *F*_(1, 31)_ = 18.05, *p* = 0.0002.

As expected, when using imagery, the participants required more time and made more errors with the complex stimuli than the simple ones and required more time and made more errors with probes that fell on “late” segments; neither of these two effects was significant in the perception condition. These findings replicate those of previous studies (e.g., Kosslyn et al., 1988) and may provide evidence that participants did in fact visualize the uppercase letters in this task. Because participants had no idea where the X would fall, and on which segment of which letter, they could not simply count in advance how many segments would be drawn to reach the location of the X before responding. Moreover, such counting would be much slower than the roughly 200-ms difference we found for near vs. far segments in the imagery task. The most parsimonious and simple explanation for the results may be that participants used mental imagery, which also can account for the longer response times when more segments had to be generated and when the X appeared in segments further away in the generation process.

#### 2.5.3 Image generation: grids

Kosslyn et al. (1995) showed that relatively minor variations of the image generation task changed which cerebral hemisphere performed the task better. In particular, participants performed the brackets task just described faster and more accurately when the stimuli were presented in the left visual field, and hence, the right hemisphere received the information initially. In contrast, when the X mark was presented not within four brackets but instead within a 4 × 5 grid, now the task was performed faster and more accurately when the stimuli were presented in the right visual field, and hence the left hemisphere received the information initially (for an account of these findings, see Kosslyn et al., 1995). Thus, we also conducted a grid version of the image generation task. This task appears to rely on spatial processing and activates the dorsal but not the ventral visual stream. Therefore, it was classified as a dorsal task (Kosslyn et al., 1985, 1995, 1998b, 2005).

##### 2.5.3.1 Method

###### 2.5.3.1.1 Materials

The materials in the Brackets task were altered in two ways: first, the participants received H and U as the simple letters (each of which had three segments) and J and S as the complex letters (which had 4 and 5 segments, respectively). Second, the letters now appeared in a 4 × 5 grid during study, and the test stimuli either contained those letters in grids along with an X (perception condition) or contained only the X in a grid (imagery condition). For an example of the stimuli used, see Figure 2.

###### 2.5.3.1.2 Procedure

The procedure was identical to that for the Brackets task.

##### 2.5.3.2 Results and discussion

###### 2.5.3.2.1 Error rates

Participants made more errors in the imagery condition than in the perception condition (3% vs. 1%), *F*_(1, 31)_ = 4.25, *p* < 0.05. In the perception condition, the participants made the same numbers of errors for the simple and complex stimuli (1% vs. 1%), F < 1; in the imagery condition, they also made comparable errors in the two conditions (2% vs. 3%), F < 1. Similarly, participants made comparable errors with early and late probes in the perception condition (1% vs. 4%), *F*_(1, 31)_ = 3.52, *p* = 0.07, and in the imagery condition (2% vs. 4%), *F*_(1, 31)_ = 2.71, *p* = 0.11.

###### 2.5.3.2.2 Response times

Participants were slower in the imagery condition than in the perception condition (1,314 vs. 712 ms), *F*_(1, 31)_ = 116.06, *p* = 0.0001. In the perception condition, participants required more time with the complex letters than the simple ones (735 vs. 689 ms), *F*_(1, 31)_ = 18.60, *p* = 0.0002. During imagery, participants also required more time for the complex letters than the simple ones (1,436 vs. 1,191 ms), *F*_(1, 31)_ = 11.94, *p* = 0.002. When probe distance was analyzed, in the perception condition, participants had equivalent response times with both early and late probes (706 vs. 714 ms), F < 1. In the imagery condition, replicating previous results, participants required more time with late than early probes (1,305 vs. 1,123 ms), *F*_(1, 31)_ = 14.81, *p* = 0.0006.

We had clear evidence that participants required more time with the more complex stimuli in the perception condition. We have no good account for this.

#### 2.5.4 Image rotation

This task assesses the participant's ability to transform objects in visual mental images. The original version of this task was developed by Shepard and Metzler (1971), and many variants have been developed (e.g., see Shepard and Cooper, 1982). In this task, participants must mentally rotate an object, “seeing it” as if it were being rotated. Previous researchers have found that the time to rotate increases systematically with the amount of rotation (Shepard and Cooper, 1982). Deutsch et al. (1988) and Cohen et al. (1996) found increased blood flow in the parietal but not temporal lobes in a mental rotation task, which provides evidence that the task draws on spatial processes (see also Crivello et al., 1996; Tagaris et al., 1997; Kosslyn et al., 1998a; Hugdahl et al., 2006).

##### 2.5.4.1 Method

###### 2.5.4.1.1 Materials

We prepared pairs of block patterns, one to the left and one to the right of a central fixation point (an asterisk). Each stimulus was composed of five squares that were connected on random sides to form an angular shape. The pattern on the right of central fixation was identical to or a mirror image of the pattern on the left. The top of each pattern was identified by a black square. Participants were asked to decide whether the shape on the right was the same as, or if it was a mirror image of, the shape on the left. Each pattern subtended a maximum visual angle of 5° horizontally and 3.6° vertically and was displaced 1.5° to the right or the left side of the central fixation point. The pattern on the right was rotated 0° in the perception condition, and thus no mental rotation was required; in the imagery condition, the pattern on the right was rotated 90° or 135°. Each degree of rotation appeared equally often with the same and different pairs. Four different types of shapes were used. For examples of the stimuli used, see Figure 3.

###### 2.5.4.1.2 Procedure

In the perception condition, an exclamation point appeared on the screen until participants pressed the spacebar. A blank screen then appeared for 500 ms, followed by the fixation point; after 500 ms, a pattern appeared to the left and another to the right of the fixation point. Participants decided whether the pattern on the right was the same as, or if it was a mirror image of, the pattern on the left.

In the imagery condition, the trial sequence was the same, but only the pattern on the left was presented upright (with the black square on the top). The pattern on the right was rotated at either 90° or 135°. Participants decided whether the pattern on the right was the same as the pattern on the left or was a mirror image of it. They were told that the shapes were identical if the one on the right could be slid over to cover the shape on the left; the shapes were not identical if the one on the right had to be flipped over before covering the one on the left.

##### 2.5.4.2 Results and discussion

###### 2.5.4.2.1 Error rates

Participants made more errors in the imagery condition than in the perception condition (9% vs. 2%), *F*_(2, 62)_ = 6.49, *p* = 0.003. In the imagery condition, there was no difference in the error rates for 90° and 135° orientations (10% vs. 9%), F < 1.

###### 2.5.4.2.2 Response times

Participants were slower in the imagery condition than in the perception condition (2,533 vs. 1,046 ms), *F*_(2, 62)_ = 113.90, *p* = 0.0001. In the imagery condition, participants required more time with the 135° rotations than with the 90° rotations (2,628 vs. 2,439 ms), *F*_(1, 31)_ = 4.13, *p* = 0.051.

In short, it appeared that our rotation task did in fact tap mental rotation.

#### 2.5.5 Motor imagery

This task is similar to the image rotation task described earlier, only the type of stimulus differs. Shepard and Cooper (1982) showed that when participants are required to rotate images of hands, they usually imagine rotating their own hands. This task was classified as spatial, given that the key factors are the orientation of the hands and a left-right judgment. Moreover, functional brain imaging studies showed that the dorsal but not the ventral pathway is activated in such tasks (Kosslyn et al., 1998a; Doganci et al., 2023).

##### 2.5.5.1 Method

###### 2.5.5.1.1 Materials

Stimuli were hands similar to those originally used by Shepard and Cooper (1982). Pairs of stimuli represented either two right hands or two left hands (“Yes” responses), or one right and one left hand (“No” responses). Each trial contained either two palms or two backs of the hands. Stimuli subtended a visual angle of 3° both horizontally and vertically and were displaced 1° to the right and to the left of a central fixation point. As in the image rotation task, the perception condition contained pairs with no rotation, and the imagery condition contained pairs with 90° or 135° of rotation. All other aspects of the design were the same as for the rotation task. Yes/No responses, the palm or back of the hands, and the combination of right/left hands were all counterbalanced. For an example of the stimuli, see Figure 4.

###### 2.5.5.1.2 Procedure

The procedure was identical to that for the image rotation task. However, in this task, the participants were told that the “standard” position of the hands was with the fingers at the top.

##### 2.5.5.2 Results and discussion

###### 2.5.5.2.1 Error rates

Participants made an equivalent number of errors in the perception and imagery conditions (2% vs. 3%), F < 1. In the imagery condition, participants made a comparable number of errors for 90° and 135° (2% vs. 3%, respectively), F < 1.

###### 2.5.5.2.2 Response times

Participants were slower in the imagery condition than in the perception condition (1,488 vs. 873 ms), *F*_(2, 62)_ = 81.05, *p* = 0.0001. In the imagery condition, participants were slower when the shapes were rotated 135° than when they were rotated 90° (1,604 vs. 1,372 ms), *F*_(1, 31)_ = 23.14, *p* = 0.0001.

The results then indicate that participants did, in fact, rotate the images in the imagery condition.

#### 2.5.6 Image scanning

Image scanning occurs when one covertly shifts attention to different parts of a visualized pattern. The task used in the present study was originally developed by Finke and Pinker (1982) and modified by Kosslyn et al. (1990a), Dror et al. (1993), and Dror and Kosslyn (1994). In the perception condition, participants see a donut-square grid and decide whether an arrow points to the center of a filled-in cell of the grid. In the imagery condition, the grid is presented very briefly, and participants need to scan a mental image to decide whether the arrow points to one of the filled cells. Previous studies have shown that participants take longer to scan greater distances across visualized objects (Kosslyn, 1973; Dror et al., 1993; Dror and Kosslyn, 1994); in this task, the further the arrow is from the target, the slower the participant will be to respond. This task was classified as spatial because of the requirement to shift attention over space to determine the location of a cell in relation to the arrow (Kosslyn, 1994; Borst et al., 2006).

##### 2.5.6.1 Method

###### 2.5.6.1.1 Materials

A donut square grid was presented at the center of the computer monitor and subtended a visual angle of 7° both horizontally and vertically. The grid was composed of six smaller squares per side, and three of the squares within this grid were filled with black. The locations of the black squares (on the top, bottom, left, or right sides of the grid), the location of the arrow within the grid (right or left visual fields, top or bottom), and the direction of the arrow (pointing up, down, left or right) were counterbalanced. The arrow was 4 mm long and appeared at one of two possible distances from the target; near arrows were located 1 cm from the inside border of the grid, whereas far arrows were located 2 cm from the inside border of the grid. Half the arrows were near, and half were far. Half the time, the arrow pointed to a square that was black, and half the time it did not. For a typical example of the stimuli, see Figure 5.

###### 2.5.6.1.2 Procedure

In the perception condition, participants pressed the spacebar, and after 500 ms, a donut square grid was presented at the center of the screen. Participants memorized the locations of the black squares and then pressed the spacebar. After 250 ms, an arrow appeared within the donut-squared grid and remained visible until a response key was pressed. Participants pressed the “Y” key if the arrow pointed to the center of one of the black squares, and the “N” key if it did not.

In the imagery condition, participants studied the location of the black squares, but this time, when they pressed the spacebar, an arrow was flashed for only 50 ms within the grid, after which both the arrow and the grid disappeared. Participants scanned their mental images to decide whether the arrow was pointing to the center of one of the black squares.

##### 2.5.6.2 Results and discussion

###### 2.5.6.2.1 Error rates

Participants made more errors in the imagery condition than in the perception condition (5% vs. 2%), *F*_(1, 31)_ = 4.64, *p* = 0.04. In both perception and imagery, participants made equivalent numbers of errors for near and far arrows; in the perception condition, 2% vs. 2%, F < 1, and in the imagery condition, 5% vs. 6%, F < 1.

###### 2.5.6.2.2 Response times

Participants were slower in the perception condition than in the imagery condition (797 vs. 714 ms), *F*_(1, 31)_ = 21.93, *p* = 0.0001. For both perception and imagery, participants were slower for the far arrows than for the near arrows: in the perception condition, 826 vs. 767 ms, *F*_(1, 31)_ = 11.11, *p* = 0.002; in the imagery condition, 738 vs. 690 ms, *F*_(1, 31)_ = 13.45, *p* = 0.0009.

Participants may have been slower in the perception condition than in the imagery condition because they spent unnecessary amounts of time encoding the arrow in the perception condition or double-checked their responses before pressing a key. In contrast, in the imagery condition, the display faded rapidly, which may have been an impetus to the participants to respond quickly. We also found the typical increased time with distance scanned. However, unlike previous results (e.g., Pinker, 1980), the magnitude of the scanning effect was comparable in both conditions. It is possible that the arrow was so short that participants needed to focus on it in both conditions, and only afterwards could shift their attention appropriately.

#### 2.5.7 Image maintenance

Image maintenance is the ability to retain imaged patterns in short-term memory. The task we used was originally developed by Kosslyn et al. (1990a), and a variant was used by Dror et al. (1993), Dror and Kosslyn (1994). Participants study the location of either two or four gray squares within a region delimited at the corners by four brackets, and then press the spacebar. They retain an image of the squares for three 3 s, and then an X appears. The participants are to decide whether the X appears in a location previously occupied by one of the squares. We classified this task as spatial because the key to correct performance is retaining the squares in the correct locations; it is location, not shape, that determines the level of performance in this task (Kosslyn et al., 1990a; Dror et al., 1993; Dror and Kosslyn, 1994; Kosslyn, 1994).

##### 2.5.7.1 Method

###### 2.5.7.1.1 Materials

We constructed stimuli by filling in 2 or 4 cells of 4 x 5 grids with gray. Afterwards, all but the corner brackets of the grid were eliminated, leaving a set of four brackets that delimited a region in which 2 or 4 squares appeared. In the perception condition, each of these stimuli was paired with another in which an X mark was added; half the time the X mark was superimposed on a square, and half the time it was next to a square. In the imagery condition, the squares were removed from the second stimulus of the pair, so only the X mark was visible. The stimuli subtended a visual angle of 4° both horizontally and vertically. The number of squares (2, 4), location of the X (right or left, up or down of midline), and location of the targeted square (left, right, up, or down) were all counterbalanced across the task. For an example of the type of stimulus used in this task, see Figure 6.

###### 2.5.7.1.2 Procedure

An exclamation point was first presented at the center of the screen. When ready, participants pressed the spacebar, and the next trial began. After 500 ms, a set of brackets appeared in the middle of the screen. Either two or four gray squares appeared inside the brackets. Participants were required to study the locations of the gray squares. After participants had memorized them, they pressed the spacebar. In perception, a blank screen was then presented for 50 ms, and was then replaced by the pattern previously studied for 2,950 ms. After that, an X appeared within the brackets, and participants decided whether the X fell on one of the gray squares.

In the imagery condition, after studying the location of the gray squares, participants pressed the spacebar, the pattern disappeared, and the participants were to retain a mental image of the locations of the squares for 3 s. At this point, the set of brackets returned to the screen with only an X inside the brackets; now, the participants decided whether the X fell on a location previously occupied by a gray square.

##### 2.5.7.2 Results and discussion

###### 2.5.7.2.1 Error rates

Participants made more errors in the imagery condition than in the perception condition (5% vs. 2%), *F*_(1, 31)_ = 7.15, *p* = 0.01. Participants made an equivalent number of errors with 2 vs. 4 squares in both perception (2% vs. 2%), F < 1, and imagery (4% vs. 6%), F < 1.

###### 2.5.7.2.2 Response times

Participants were slower in the imagery condition than in the perception condition (1,221 vs. 720 ms), *F*_(1, 31)_ = 92.61, *p* = 0.0001. In the perception condition, participants had equivalent response times with 2 vs. 4 squares (717 vs. 723 ms), F < 1. In the imagery condition, however, participants were slower with 4 squares than 2 (1,323 vs.1,120 ms), *F*_(1, 31)_ = 13.18, *p* = 0.001.

As expected, participants were slower in the imagery condition when more material had to be retained. The lack of an effect of the number of squares in the perception condition shows that the effect was not due to searching for a square when the X appeared. Thus, we can be confident that the imagery task does indeed tap image processing *per se*.

#### 2.5.8 Spatial imagery

We designed this task to assess the representation of relative locations. In the perception condition, participants saw a 2 x 2 grid with a filled circle at the center. They then heard a set of directions, and at the same time saw the circle moving along these directions. Participants then heard a cue word (above, below, right, or left) and decided whether it correctly described the circle's final location relative to its initial location in the grid. In the imagery condition, participants heard the same set of directions, but this time visualized the circle traveling along the stated directions on an imaginary grid. When they heard the cue word, they decided whether it correctly described the final location of the circle relative to its initial location. We classified this task as spatial because of the encoding of different spatial directions and because of the directional judgment that participants had to make. Moreover, functional brain imaging studies suggest that similar tasks activate the parietal but not the temporal lobes (Zeki, 1990a; Zeki et al., 1991; Beckers and Homberg, 1992; Zeki et al., 1993; Watson et al., 1993; Sereno et al., 1995; Shipp et al., 1995; Tootell et al., 1995a,b).

##### 2.5.8.1 Method

###### 2.5.8.1.1 Materials

A 2 x 2 inch grid was presented at the center of the screen, with a filled circle at the center of the grid. The diameter of the filled circle was 6 mm. In the perception condition, a set of directions was recorded and presented by the computer, one at a time when the spacebar was pressed. Along with each word, the circle shifted to the appropriate position. At the end of 4, 5, 6, or 7 directions (on an equal number of trials), the participants heard the word right, left, above, or below, and the computer recorded the time for them to respond. The imagery trials were the same except that only the direction words were presented. The grid subtended a visual angle of 5.7° both horizontally and vertically. We varied the number of directions between trials to avoid the possibility that participants disregarded the first few trials and to encourage them to pay attention throughout the task. The number of directions and the directions in which the circle moved (east, west, north, south, southeast, southwest, northeast, and northwest) were balanced across trials. For an example of the stimuli used in this task, see Figure 7.

###### 2.5.8.1.2 Procedure

In the perception condition, each trial began with a fixation point, and 500 ms after participants pressed the spacebar, they heard the first direction in the set while they saw the filled circle moving at constant speed from one location to another in the stated direction. After the circle had moved, the participants pressed the spacebar to cause the circle to move in a new direction. After the last direction was spoken and the circle moved to the final location, the participant heard one of four cue words and decided whether the word accurately described the circle's final location relative to its initial location on the grid. If the word accurately described the relation, the participant pressed the “Y” key; if it did not, he pressed the “N” key.

In the imagery condition, the participants heard a similar set of directions, but this time were asked to visualize the circle moving in the stated directions along an imaginary grid. All other aspects of the procedure were the same as in the perception condition.

##### 2.5.8.2 Results and discussion

###### 2.5.8.2.1 Error rates

Participants made more errors in the imagery condition than in the perception condition (8% vs. 4%), F _(1, 31)_ = 5.66, *p* = 0.02. When we analyzed the effect of the number of directions (short or long, with 4 and 5 or 6 and 7 directions respectively), we found that it was non-significant for both perception, 5% vs. 3%, F_(1, 31)_ = 1.00, *p* = 0.33, and imagery (8% vs. 8%, F < 1).

###### 2.5.8.2.2 Response times

Participants had slower response times in the imagery condition than in the perception condition (1,323 vs. 1,188 ms), F_(1, 31)_ = 5.95, *p* = 0.02. When we analyzed the effect of the number of directions (short vs. long) we found that in both perception, F_(1, 31)_ = 5.69, *p* = 0.02, and imagery, F_(1, 31)_ = 7.11, *p* = 0.01, participants required more time for directions of different lengths. However, surprisingly, the effect was not in the expected direction (with means of 1,229 and 1,150 ms for short and long trials in the perception condition, and with means of 1,378 and 1,259 ms for short and long trials in the imagery condition).

We do not know why we found significantly longer response times when the number of directions was short rather than long; we expected the opposite. However, in light of the results, it is possible that participants took more time for the short trials because they did not know when the trial was going to end, and because some trials were long, they may not have been ready to respond when the short trials ended. Alternatively, in the long trials, participants knew that there would be a high likelihood of the trial ending soon, and they were therefore ready to respond.

#### 2.5.9 Object imagery

We posited that this and the following three tasks rely on the ventral system. The first task is a modification of one developed by Eddy and Glass (1981). Participants heard statements about properties of common objects and decided whether they were true or false; the statements were constructed so that participants needed imagery to evaluate them if no picture was presented. In the imagery condition, the statements were presented in isolation; in the perception condition, pictures of the named objects were presented along with the statement. Goldenberg et al. (1989), using single photon emission computed tomography, showed that similar tasks activate the temporal but not the parietal lobes. Similarly, Fletcher et al. (1995), Beauregard et al. (1997), and D'Esposito et al. (1997) found activation of the temporal but not the parietal lobes in similar tasks. Thus, we can assume that this is a “ventral” task.

##### 2.5.9.1 Method

###### 2.5.9.1.1 Materials

For the perception condition, we prepared 16 line drawings of objects or animals. Line drawings were derived from the Snodgrass and Vanderwart (1980) set, and their sizes were adjusted so that all pictures subtended a maximum visual angle of 7° both vertically and horizontally. Each drawing was paired with an auditorily presented statement that described a subtle visual feature or property of that object or animal. The statements were recorded in a male voice and stored on the computer. Half of the statements were true, and half were false. In the imagery condition, a similar set of statements was presented; half the participants received one set of statements in the imagery condition, and the other in the perception condition, and vice versa for the other half.

We determined the best items for this task by collecting ratings from 10 right-handed Harvard undergraduates (five males and five females), between the ages of 18 and 24, who were given a list of 40 short sentences that described a particular feature of an object (20 items) or animal (20 items). Participants were asked to judge whether the sentence was true or false, and to rate the amount of imagery they used to make their judgment on a scale of 1-7 (1 = very little imagery; 7 = a large amount of imagery). For the perceptual component of the experiment, 40 line drawings were centered in a 6.2 cm × 6.2 cm area (7° visual angle) on the computer screen. A second group of 10 Harvard undergraduates between the ages of 18 and 24 was then given a list of the same 40 sentences that the first group of participants was given and was asked to judge whether each statement was true or false based only on the information provided in the corresponding line drawing.

We only included statements that at least 80% of the participants agreed on the validity; of these, we used the 16 highest rated imagery sentences about animals and the 16 highest rated sentences about objects. Two equivalent sets of items were created, which did not differ in the amount of imagery involved (*p* > 0.05), and each version included the same number of objects and animals. For examples of the type of stimuli used in this test, see Figure 8.

###### 2.5.9.1.2 Procedure

In the perception condition, an exclamation point appeared at the center of the screen; 500 ms after the participants pressed the spacebar, a line drawing was presented along with a statement that described a particular feature of the object or animal. In the imagery condition, only the sentence was presented. Participants decided, either based on the picture or a mental image, whether the statement was true or false.

##### 2.5.9.2 Results and discussion

###### 2.5.9.2.1 Error rates

Participants made an equivalent number of errors in the perception and imagery conditions (6% vs. 9%), F_(1, 31)_ = 1.68, *p* = 0.21.

###### 2.5.9.2.2 Response times

Participants were slower in the imagery condition than in the perception condition (2,874 vs. 2,669 ms), F_(1, 31)_ = 21.71, *p* = 0.0001.

The results were similar to those of Eddy and Glass (1981), who used interference techniques to demonstrate that imagery was, in fact, used when participants had to evaluate these types of statements.

#### 2.5.10 Face Imagery

Participants decided which of two famous faces was rounder. Jolicoeur and Kosslyn (1983) provided evidence that subtle, relatively unfamiliar spatial judgments are performed using imagery. This task was classified as ventral because Goldenberg et al. (1989), Haxby et al. (1991), Haxby et al. (1994), Andreasen et al. (1996), Clark et al. (1996), Puce et al. (1996), and Kanwisher et al. (1997) demonstrated that judgment of facial features is associated with increases in blood flow in the temporal but not parietal lobes. In addition, disruption of face processing is clearly associated with damage to the ventral system (Farah, 2004).

##### 2.5.10.1 Method

###### 2.5.10.1.1 Materials

We paired photographs of the faces of famous people. The photographs included politicians, actors, and singers (e.g., Chaplin, Clinton, and Hitler), and they depicted the faces in front view, facing slightly to the right or the left, but with all facial features visible. The photographs were gathered from biographies of famous people and assorted news magazines and were scanned into Adobe Photoshop using a 7700 Epson Ecotank scanner. Photographs were chosen according to ratings of familiarity from an independent participant sample. Specifically, we began by collecting ratings from 26 Harvard undergraduates (13 males/13 females) between the ages of 18 and 24, who were given a list containing the names of 140 famous people. Participants indicated whether or not they could visualize the face of each person on the list. Notably, 24 famous males and 15 famous females were selected from this list based on the number of participants who could image the faces (which ranged from 83% to 100%). Because of the low familiarity of most of the female faces on the list, nine new famous female faces were added to the list. They were female faces often mentioned as familiar by the participants who performed the ratings task.

In a second round of ratings, 16 right-handed Harvard undergraduates (eight males/eight females), ranging in age from 19 to 24, participated in a perceptual and an imagery version of the task. The order of the condition (imagery first or perception first) was counterbalanced across all participants. Participants were presented with 120 pairs of faces with the 24 best-known males and 24 best-known females from the prior ratings task. Pairs consisted of either two male or two female faces judged by the examiners to be very close in roundness.

In the perceptual version of this ratings task, participants were shown black and white photographs of pairs of faces presented on the computer screen. Participants heard the names of the two famous people, one after another, and at the same time, a photograph of their faces appeared on the computer screen. The first person's face always appeared on the left-hand side of a center line, in a region of 6.2 × 6.2 cm on the computer screen, while the second person's face appeared on the right-hand side within a similar region, each face subtending a 7° visual angle. If the first face was rounder than the second, participants pressed the “Y” key; if it was not, they pressed the “N” key. Both faces remained on the screen until participants made a judgment as to whose face was rounder. Participants then rated on a scale of 1 to 7 how close the two faces were in roundness (with 1 = not very close and 7 = very close).

In the imagery version of this ratings task, participants heard the names of two famous people, separated by 2 s. Participants formed a mental image of each person's face and decided which of the two faces was rounder. Participants were instructed that “roundness” should be determined by how circular or full the shape of a face was. Again, if the first face was rounder than the second, participants pressed the “Y” key; if it was not, they pressed the “N” key. After making this judgment, participants rated on a scale of 1-7 how close the two faces were in roundness (with 1 = not very close, and 7 = very close).

After participants performed both the imagery and the perceptual ratings tasks, they were given a questionnaire that asked them to rate on a scale of 1-7 the familiarity of each person's face and how well the photographs of the person matched their mental image of the person's face.

Results from this rating analysis were used in the final selection process. All final test pairs had ratings that did not differ significantly in the perception and imagery conditions (*p* > 0.05). A minimum response accuracy of 70% was required in both imagery and perception. The 16 pairs were then divided into two sets of 8 (four females/four males in each set). We selected pairs so that the two versions were equivalent, taking into account the closeness ratings (Version 1: 3.74 ± 0.90, range: 2.44-5.00 for perception and 3.62 ± 0.59, range: 3.00-4.73 for imagery; Version 2: 3.62 ± 0.52, range: 2.63-4.19 for perception and 3.64 ± 0.39, range: 2.88-4.00 for imagery), the matching of the mental image ratings to the perceptual analog (5.37 ± 0.71 for Version 1 and 5.40 ± 0.80 for Version 2), and the familiarity ratings (5.33 ± 0.74 for Version 1 and 5.28 ± 1.00 for Version 2). Faces subtended a visual angle of 7° horizontally and 7° vertically and were presented to the right or to the left of midline. Each pair of pictures was accompanied by digitized recordings of the names of the people.

In both conditions, each pair of faces was repeated two times because of the small number of famous faces known by a large proportion of participants. The first time, Face 1 was paired with Face 2, and the second time, Face 2 was paired with Face 1. A repeated pair of faces was not presented until all faces had been presented once. Half the photographs were female and half were male faces; half the time, female faces were compared with male faces, and the other half were presented with female faces. The opposite was true for male faces. We constructed two sets, which were counterbalanced over imagery and perception conditions. A minimum response accuracy of 70% had to be reached for an item to be included in the final version of the test (i.e., 70% of the participants had to agree that a particular face was rounder than the other. Versions 1 and 2 were comparable in terms of both response accuracy and difficulty of the discriminations).

###### 2.5.10.1.2 Procedure

In the perception condition, a fixation point was presented until participants pressed the spacebar. After 500 ms, two famous faces were presented side by side, first the one on the left and then, 500 ms later, the one on the right. Simultaneously, the names of the people were read aloud by the computer. In the imagery condition, the participants heard the names of two people, presented 500 ms apart, and were asked to visualize the faces. In both conditions, the participants were to decide whether the first face was rounder than the second. After the response, the fixation point was presented until participants pressed the spacebar. After 500 ms, the next pair was presented.

##### 2.5.10.2 Results and discussion

###### 2.5.10.2.1 Error rates

Participants made more errors in the imagery condition than in the perception condition (23% vs. 11%), F_(1, 31)_ = 22.55, *p* = 0.0001.

###### 2.5.10.2.2 Response times

Participants were slower in the imagery condition than in the perception condition (2,022 vs. 1,315 ms), F_(1, 31)_ = 51.65, *p* = 0.0001.

These results are consistent with the inference that participants used imagery; the participants generally also reported having used imagery.

#### 2.5.11 Color imagery

In this task, participants determined the validity of a statement about the color of an object, either based on a picture or a mental image. Because the statements referred to subtle, relatively unfamiliar properties, we expected imagery to be used (see Jolicoeur and Kosslyn, 1983). Moreover, because color discriminations activate regions of the ventral system, namely the temporal lobes (Lueck et al., 1989; Zeki, 1990b; Zeki et al., 1991; Corbetta et al., 1991; Zeki et al., 1993), we classified the color imagery task as ventral.

##### 2.5.11.1 Method

###### 2.5.11.1.1 Materials

Stimuli were close-up photographs of typical food items of one of four colors: red (four trials), green (four trials), yellow (four trials), or brown (four trials). Typical items were a watermelon, a banana, or parsley, which were photographed against a gray background, and then transferred into the computer and cropped. If needed, color balance was adjusted. The photographs subtended a maximum visual angle of 7° both horizontally and vertically. Sentences were spoken by the computer speaker in a male voice.

We selected the items for this task after two rounds of ratings by independent groups of participants. In the first round, 16 participants, half of each gender (age range 18-24), judged the validity of each of 280 sentences. The named objects were green, red, yellow, or brown. In the perception condition, participants saw one item at the center of the computer screen at a 7° visual angle and simultaneously heard a sentence. They decided whether the sentence was true or false based on the picture they saw on the screen. Participants then rated on a scale of 1 (= easy) to 7 (= difficult) how difficult it was to determine the validity of the sentence. Following that, the next object was presented. In the imagery condition, participants received a list of the sentences on paper and visualized each named object. Participants were instructed to visualize the most prototypical colors of the objects and to judge the validity of the sentence. Participants rated the difficulty of determining the validity of the sentence on a scale of 1-7, and also rated how much they used imagery for each sentence on a scale of 1 (= no imagery) to 7 (= strong imagery).

We then calculated the percent response accuracy for each item in the test separately for perception and imagery ratings. Only items with a minimum response accuracy of 65% were used in the second round of ratings. Two sets of 20 sentences each were selected, and *t*-tests were used to determine that Version 1 and Version 2 were comparable in difficulty and response accuracy. All *t*-tests had a *p* > 0.05. Paired *t*-tests were calculated on the difference between the mean difficulty rating in the perception condition vs. the difficulty of the same item in the imagery condition. Only items that had a similar level of difficulty (with *p* > 0.05) were considered for the final version of the task.

In the second round of ratings, we only administered the perceptual version of the task. Participants rated a list of 70 objects presented centrally on the computer screen, each of which subtended a 7° visual angle. Participants rated the items for familiarity on a scale of 1 (not familiar at all) to 5 (very familiar) and rated how well the colored pictures compared with their mental image of the objects on a scale of 1 (very different) to 5 (very similar). For scores 1, 2, and 3, participants were asked to describe how the actual picture differed from their own mental images. Only images with similarity scores >4 were used for the actual experiment.

After the second round of ratings, we again ensured that accuracy was at least 65%. The best 40 sentences were used for the final test. The two versions of the test had four practice and 16 test trials each. We chose the final test items based on a high degree of familiarity for all participants, and also with a high degree of matching between the perception test items and the actual mental images (all means > 4). The sentences described the color of the food item with a qualifier such as “light” and “dark” (Version l) or “pale” and “deep” (Version 2). For example: “parsley is dark green”; or “the inside of a banana is deep yellow.” Qualifiers were balanced across participants and across conditions.

###### 2.5.11.1.2 Procedure

A central fixation point was presented until the participant pressed the spacebar. In the perception condition, after 500 ms, a picture of a food item was presented at the center of the computer screen, and simultaneously, a sentence describing the color of the object was presented. In the imagery condition, participants heard the sentence describing the color of the food item, but no photograph was presented. Participants were asked to decide, either based on the photograph or a mental image, whether the sentence was true or false. The next stimulus was presented after a response was entered.

##### 2.5.11.2 Results and discussion

###### 2.5.11.2.1 Error rates

Participants made more errors in the imagery condition than in the perception condition (26% vs. 9%), F_(1, 31)_ = 72.00, *p* = 0.0001.

###### 2.5.11.2.2 Response times

Participants were slower in the imagery condition than in the perception condition (2,572 vs. 2,191 ms), F_(1, 31)_ = 20.80, *p* = 0.0001.

These results are consistent with the inference that participants used imagery. Indeed, participants reported having used imagery.

#### 2.5.12 Auditory imagery

Participants decided whether the first of two successively presented sounds (or sounds made by named objects or animals, in the imagery condition) was higher in pitch than the second. Because regions of the temporal lobes have been activated in similar tasks (Mazziotta et al., 1982; Patterson et al., 2002; Griffiths, 2003; Bendor and Wang, 2005; Alho et al., 2014), this task was treated as a ventral task. However, this classification must be considered with caution, given that the task does not rely on vision, but on hearing.

##### 2.5.12.1 Method

###### 2.5.12.1.1 Materials

In the perception condition, we paired two-second segments of sounds obtained from commercial CDs. They included common sounds of animals and objects. Sounds were presented one following another and were paired based on preliminary ratings of similarity and a minimum response accuracy of 70% in an independent group of participants. We began to construct items by collecting ratings from 16 right-handed Harvard undergraduates (eight males and eight females), age 18-24, who rated a list of 126 items for pitch. Half the participants rated the items using imagery first, and the other half rated them perceptually first. In the perception condition, participants heard digitized sounds, presented one at a time for 2 s each, with an intertrial interval (ITI) of 2 s, and rated the pitch of each sound on a scale of 1 to 7 (with 1 = low pitch; 7 = high pitch). Participants also rated how easy it was to determine the pitch of each sound (with 1 = easy; 7 = difficult). In the imagery condition, participants heard the names of the same sounds presented one at a time (with a 2 s ITI) and had to evaluate the pitch of each sound based on a mental image of the sound. The order of the conditions was counterbalanced across participants. Items that had at least 70% minimum response accuracy in both perception and imagery were included. We also selected the items based on their lower variances: whereas some items were rated along the entire range of the scale 1-7 (reflecting that although for some participants a sound was very high in pitch, for others it was very low), other items showed more restricted ranges across participants (e.g., ranging from 1 to 4), indicating that an item was consistently rated as high (or low) pitched across different participants. We chose the sounds that were associated with lower variances in their ratings across different participants. We paired the sounds and constructed two alternate versions in which all pairs of items were similar. The difficulty ratings did not differ in the perception and imagery conditions for the same items, and items in Version 1 were not different in either similarity or accuracy from items in Version 2 (all *p* > 0.05).

In the second ratings task, we asked 16 right-handed Harvard undergraduates (eight males and eight females), ages 19-24, who had not participated in the first ratings task, to decide which of two sounds was higher in pitch. For this ratings task 83 pairs of sounds were rated in both perception and imagery conditions. Conditions were counterbalanced across participants. Participants decided which of the two sounds was higher in pitch, and rated on a scale of 1-7 the closeness of the discrimination (with 1 = close; 7 = far).

Based on the results of these ratings, we included pairs with a minimum response accuracy of 70% in both the perception and imagery conditions. Mean difficulty for each item did not differ in the perception and imagery conditions and also did not differ across Versions 1 and 2 (paired *t*-tests, all *p* > 0.05). Mean closeness of the items for the final versions of the test was 3.866 ± 0.61 in the perception condition (range: 2.75-4.69) and 3.789 ± 0.72 in the imagery condition (range: 2.25-4.88) for Version 1 and 3.648 ± 1.02 (range 2.19-6) in the perception condition and 3.734 ±0.60 (range: 2.81-4.75) in the imagery condition for Version 2. For half the pairs, the first item had a higher pitch, and for the other half, the second item had a higher pitch. A minimum response accuracy of 70% was required in both conditions, and in both Versions 1 and 2. The two versions of the task did not differ in terms of the difficulty of the discriminations (*p* > 0.05). In the imagery condition, the sounds were replaced by the names of the objects, which were also presented auditorily.

###### 2.5.12.1.2 Procedure

In the perception condition, an exclamation point was presented centrally until participants pressed the spacebar. Participants then heard a stimulus pair, with a 500 ms interval between each sound. Participants decided whether the first sound was higher in pitch than the second. After a response was entered, the exclamation point returned to the computer screen. In the imagery condition, participants heard the names of two animals or objects spoken aloud (delivered by the computer), 500 ms apart, and were asked to “imagine in their mind's ear” whether the sound emitted by the first object was higher in pitch than that emitted by the second object.

##### 2.5.12.2 Results and discussion

###### 2.5.12.2.1 Error rates

Participants made more errors in the imagery condition than in the perception condition (24% vs.13%), F_(1, 31)_ = 34.93, *p* = 0.0001.

###### 2.5.12.2.2 Response times

Participants were slower in the imagery condition than in the perception condition (2,693 vs. 2,121 ms), F_(1, 31)_ = 13.35, *p* = 0.0009.

Again, participants reported using imagery when interviewed afterwards. Thus, we have some reason to believe that imagery was used in the imagery condition.

#### 2.5.13 Word imagery

The following three tasks were classified as relying on a mixture of dorsal and ventral processing. Ventral processing is used to retrieve the objects to be compared from long-term memory, and dorsal processing is used in making the judgments, which, in the following three tasks, require spatial information.

The word imagery task is a modification of a task originally designed by Weber and Castelman (1970) and Weber and Malmstrom (1979). It was classified as relying partly on ventral processing because the judgment concerns a particular property of the appearance of words, which is represented in the ventral system (e.g., Petersen et al., 1988; Cohen and Dehaene, 2009; Zhou et al., 2016). It was classified as relying partly on dorsal processing because participants were asked to judge the relative heights of letters in the words, which is clearly a spatial judgment.

##### 2.5.13.1 Method

###### 2.5.13.1.1 Materials

For this task, 24 low-imagery, high-frequency (Thorndike and Lorge, 1966) four-letter verbs were chosen (e.g., live, wish). The first and the last letters of each word were either both tall (such as in “hold,” four trials), both short (such as “move,” four trials), the first was tall and the last short (such as “live,” four trials), or the first was short and the last tall (such as “meet,” four trials). In the perception condition, words were presented centrally on the screen in a black 24-point Chicago plain font on a white background and subtended a visual angle of 3.4° horizontally and 1.2° vertically. In the imagery condition, the words were read aloud by the computer. For an example of the stimuli used in this task, see Figure 9.

###### 2.5.13.1.2 Procedure

A central fixation point was presented until participants pressed the spacebar. After that, a blank screen was presented for 500 ms, followed by a stimulus. In the perception condition, a word appeared at the center of the screen. The word remained on the screen until the participant decided whether the first and last letters were the same height. In the imagery condition, the word was spoken aloud, and the participants were to visualize the word and decide whether the first and the last letters were the same height. The exclamation point returned after a response, which signaled the beginning of a new trial.

##### 2.5.13.2 Results and discussion

###### 2.5.13.2.1 Error rates

Participants made comparable numbers of errors in the two conditions (6% vs. 4% for imagery vs. perception, respectively), F_(1, 31)_ = 1.29, *p* = 0.26.

###### 2.5.13.2.2 Response times

Participants had slower response times in the imagery condition than in the perception condition (1,758 vs. 941 ms), F_(1, 31)_ = 394.69, *p* = 0.0001.

Again, our results and the participants' reports suggest that imagery was indeed used.

#### 2.5.14 Size imagery

This task required participants to judge the relative size of a pair of objects, and size is clearly represented in the dorsal system (Oliver and Thompson-Schill, 2003). However, because it involves a comparison between pairs of objects, and the shapes of objects are represented in the ventral system (Malach et al., 1995; James et al., 2003; Wilson and Wilkinson, 2015), we classified this task as relying on a mixture of ventral and dorsal processing (for a justification, see the brain activations reported Zachariou et al., 2014; Ayzenberg and Behrmann, 2022).

##### 2.5.14.1 Method

###### 2.5.14.1.1 Materials

We prepared pairs of items, which were either both animals (four trials), both objects (four trials), or an animal and an object (eight trials). The visual stimuli were line drawings of objects and animals derived from the Snodgrass and Vanderwart (1980) set. Pairs of items were depicted at appropriate relative sizes, as assessed by ratings of relative height that were obtained from an independent participant sample; ratings were obtained on the pairs (e.g., motorcycle-sheep) to produce a minimum response accuracy of 70%. We began by collecting ratings from 16 right-handed Harvard University undergraduates (eight males and eight females) between the ages of 18 and 24, who served as paid volunteers. Ratings were obtained in the imagery condition only, and the estimated size differences between pairs were then used to produce comparable perceptual items. Participants were given a list of 126 names of common objects and animals, asked to visualize each item and estimate the height of each at its tallest point. Participants were encouraged to use a yardstick for their estimates. Mean height for each item was obtained, and the 32 “closest” possible pairs of items were formed by calculating ratios among all pairs.

The perceptual and imagery versions of the task were matched as to the closeness of the pairs and accuracy (*p* > 0.05). We prepared two sets of items, which were comparable in response accuracy and the difficulty of the discriminations. Mean “closeness” of the pairs in Version 1 was 0.75 ± 0.08 (range: 0.52-0.87), and mean height of the pairs was 19.40 ± 20.85 (range: 1.10-69.41). Mean closeness of the pairs in Version 2 was 0.74 ± 0.09 (range: 0.59-0.89), and mean height of the pairs was 17.07 ± 19.91 (range: 0.91-74.85). Mean closeness and mean height of the pairs did not differ between Versions 1 and 2 (all *p* > 0.05). Stimuli subtended a maximum visual angle of 7° to the right or to the left of a central fixation point. In both conditions, participants heard the names of the objects. In the imagery condition, only the names were presented. For an example of the stimuli, see Figure 10.

###### 2.5.14.1.2 Procedure

A central fixation point was presented until the participant pressed the spacebar. After 500 ms, a stimulus was presented. In the perception condition, the stimulus was a line drawing of an object or animal presented to the left of a central fixation point on the computer monitor. At the same time, participants heard its name read aloud by the computer. After 500 ms, the second picture in the pair was presented to the right side of the central fixation point, and its corresponding name was read aloud. In the imagery condition, participants heard the name of the first object or animal, and after 500 ms, they heard the name of the second object or animal; participants were asked to visualize the objects or animals at a typical size and orientation. In both conditions, participants decided whether the first item was taller than the second item at its highest point.

##### 2.5.14.2 Results and discussion

###### 2.5.14.2.1 Error rates

Participants made more errors in the imagery condition than in the perception condition (24% vs. 5%), F_(1, 31)_ = 88.47, *p* = 0.0001.

###### 2.5.14.2.2 Response times

Participants were slower in the imagery condition than in the perception condition (2,322 vs. 1,028 ms), F_(1, 31)_ = 88.15, *p* = 0.0001.

Thus, we have some reason to infer that they did, in fact, use imagery in the imagery condition.

#### 2.5.15 Tactile imagery

In this task, participants heard the names of two common objects or animals and decided whether the first was firmer in touch than the second. We classified this task as mixed. In fact, although sensory integration regions in the parietal association areas have been shown to participate in the analysis of tactile stimuli (Roland and Mortensen, 1987; Uhl et al., 1994; Prater et al., 2004), participants also must retrieve images of objects from long-term memory, which relies on processing in the ventral system.

##### 2.5.15.1 Method

###### 2.5.15.1.1 Materials

Participants heard the names of two objects and decided whether the first object was firmer than the second. This task did not have a perception control condition, and the comparisons were either “far” (control condition, example: bear—rock) or “near” (imagery condition, example: banana—toothpaste). Kosslyn et al. (1977) showed that when participants compared the sizes of objects of very different sizes, they did not use imagery; in contrast, when they compared objects of similar sizes, they did use imagery. We expected this principle to generalize to the tactile domain.

The task included 24 pairs of items, half of which required far discriminations and half of which required close discriminations. Close and far trials were determined by the experimenters. The names of all pairs were presented aloud by the computer in a male voice. After all 24 pairs were presented once, they were all presented a second time in a different order. However, the member that was previously presented as the first item was now presented as the second one, and vice versa. For each pair, participants decided whether the first item was firmer than the second; if it was, they pressed the “Y” key, and if it was not, they pressed the “N” key. Half of the far discriminations were “Yes” responses, and the other half were “No” responses. The same rule applied to the close discriminations.

###### 2.5.15.1.2 Procedure

Participants saw an exclamation point at the center of the screen; 500 ms after they pressed the spacebar, a word was presented auditorily, followed 500 ms later by a second word. Participants decided whether the object named by the first word was firmer than the second. After a response key was pressed, the exclamation point returned to the screen to signal the beginning of a new trial.

##### 2.5.15.2 Results and discussion

###### 2.5.15.2.1 Error rates

Participants made more errors in the imagery condition than in the control condition (41% vs. 7%), F_(1, 31)_ = 107.52, *p* = 0.0001.

###### 2.5.15.2.2 Response times

Participants were slower in the imagery condition than in the control condition (2,126 vs. 1,583 ms), F_(1, 31)_ = 45.25, *p* = 0.0001.

In post-session debriefing, the participants reported that they did follow instructions and mentally “squeezed” the objects when making decisions in the imagery condition.

## 3 Multivariate analyses results

As noted, we had reason to believe that the participants did, in fact, use imagery in all the imagery tasks. Thus, it is of interest to compare the structure of processing in the imagery and perception conditions, using multivariate statistical analyses. We used several types of multivariate analyses to investigate the structure underlying the response times and error rates in the different tasks in the imagery vs. perception conditions. These analyses produced convergent results, as described below.

### 3.1 Multidimensional Scaling analyses

We used nonmetric Multidimensional Scaling (MDS) to determine whether the IPB tasks did, in fact, group into dorsal, ventral, and mixed tasks. If so, the data should form clearly delineated corresponding clusters. Tasks that rely on similar underlying mechanisms should cluster together more than tasks that rely on different mechanisms. We began by calculating separate Pearson correlation matrices for the mean response times for all pairs of tasks in the perception conditions and the imagery conditions separately. The correlation matrices for the response times are shown in Tables 2a, b. We then used MDS to scale these two correlation matrices. The higher the correlation between two tasks, the smaller the distance should be in Euclidean space. We did not analyze error rates further because many tasks had mean error rates of < 5%, making interpretation of the results difficult and inappropriate (see Table l).

**Table 2A:** Correlation matrix between response times in perceptual tasks.

**IPB task**	**1**.	**2**.	**3**.	**4**.	**5**.	**6**.	**7**.	**8**.	**9**.	**10**.	**11**.	**12**.	**13**.
1. Face													
2. Object	0.462												
3. Spatial	−0.064	0.151											
4. Tactile	0.437	0.255	0.076										
5. Rotation	0.216	0.342	0.111	0.101									
6. Word	−0.123	0.032	0.227	0.388	0.273								
7. Motor	0.180	0.184	0.397	0.151	0.585	0.545							
8. Color	0.659	0.638	0.199	0.249	0.233	−0.090	0.171						
9. Maint.	0.179	0.298	0.428	0.153	0.462	0.535	0.688	0.294					
10. Auditory	0.462	0.479	0.134	0.278	0.194	−0.127	0.131	0.451	0.302				
11. Grids	0.259	0.267	0.335	0.422	0.491	0.576	0.609	0.210	0.791	0.359			
12. Brackets	0.232	0.234	0.094	0.484	0.320	0.602	0.440	0.148	0.381	0.208	0.620		
13. Size	0.494	0.221	0.045	0.615	0.331	0.388	0.378	0.203	0.382	0.413	0.605	0.678	
14. Scanning	0.079	0.236	0.130	0.187	0.568	0.385	0.397	0.163	0.477	0.027	0.353	0.222	0.259

**Table 2B:** Correlation matrix between response times in imagery tasks.

**IPB task**	**1**.	**2**.	**3**.	**4**.	**5**.	**6**.	**7**.	**8**.	**9**.	**10**.	**11**.	**12**.	**13**.
1. Face													
2. Object	0.212												
3. Spatial	0.089	−0.001											
4. Tactile	0.475	0.088	0.335										
5. Rotation	0.159	0.443	0.257	−0.127									
6. Word	−0.019	0.366	0.291	0.112	0.017								
7. Motor	0.407	0.057	0.603	−0.035	0.529	−0.072							
8. Color	0.022	0.113	0.129	−0.053	0.067	−0.037	0.109						
9. Maint.	−0.052	0.267	0.507	−0.023	0.284	0.578	0.271	−0.209					
10. Auditory	0.456	0.449	0.232	0.302	0.177	0.373	0.156	0.419	0.251				
11. Grids	−0.117	−0.008	0.435	0.078	0.349	0.434	0.232	0.075	0.512	0.128			
12. Brackets	0.098	0.238	0.540	0.241	0.506	0.337	0.348	0.069	0.358	0.275	0.722		
13. Size	0.446	0.517	0.261	0.290	0.351	0.341	0.288	0.300	0.344	0.845	0.190	0.420	
14. Scanning	0.165	0.301	0.404	0.059	0.475	0.336	0.431	−0.076	0.386	0.075	0.414	0.605	0.177

#### 3.1.1 Response times in the perceptual conditions

**Figure 11:**
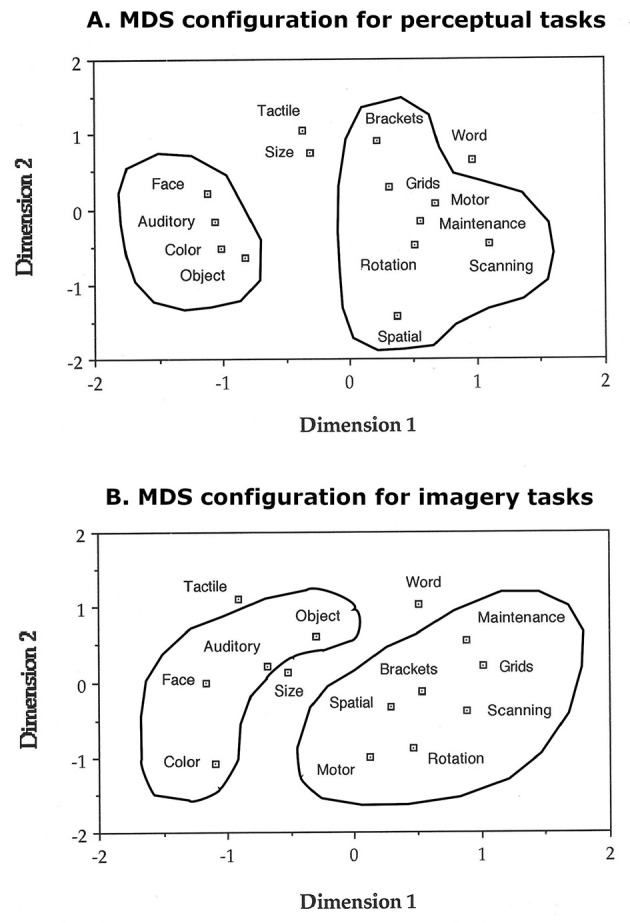
MDS configuration for perceptual and imagery tasks. For both perceptual and mental imagery tasks, there are two main groups, reflecting dorsal and ventral visual processing. A third group includes mixed tasks in perception (tactile and size). The word imagery task, which we classified as mixed, tends to cluster with dorsal tasks in perception. In the imagery condition, mixed tasks tend to cluster with either ventral tasks (tactile and size) or with dorsal tasks (word). **(a)** MDS configuration for perceptual tasks. **(b)** MDS configuration for imagery tasks.

The two-dimensional solution accounted for 89% of the variance (Figure 11a). The stress value was 0.13 after 20 iterations. This value is below the criteria for the 0.05 confidence level of the distribution of stress for scaling 12 stimuli in a two-dimensional Euclidean space (for *p* < 0.05, the necessary stress value = 0.211). This criterion was slightly more stringent than needed, given that we had 14 rather than 12 stimuli; our stress value is therefore significantly better than it would be if the data were random (see Klahr, 1969) and does not justify a higher-dimensional space.

Visual inspection of Figure 11a reveals two major clusters of tasks: the first cluster, on the positive side of Dimension 1, included the perceptual conditions in the image generation (grids and brackets), image maintenance, image rotation, motor imagery, spatial imagery, and image scanning tasks. These tasks all rely on crucial processing in the dorsal system, and all involve a spatial judgement. The word imagery task, which we had classified as requiring mixed dorsal/ventral processing because it involves objects (the words) and a spatial judgment (comparing letter heights), clustered with the spatial tasks. The second cluster, on the negative side of Dimension 1, included the perceptual conditions of the face imagery, object imagery, color, and auditory imagery tasks. A third subgroup, more to the center of Dimension 1, included the size and tactile imagery tasks that were classified as “mixed” ventral/dorsal. We therefore labeled the first dimension as the degree of “ventral-dorsal” processing required by each task.

We interpreted the second dimension as reflecting the degree of complexity of the stimuli. As can be observed in the figure, the spatial, scanning, rotation, and the color and object tasks were the most complex: complex visual stimuli as well as entire sentences, had to be processed at the low end of Dimension 2. At the upper end of Dimension 2, only pairs of words had to be processed, as in the tactile and size tasks, or simple visual stimuli (such as letters in the grids and brackets tasks, or single words in the word imagery task) had to be processed.

#### 3.1.2 Response times in the imagery condition

The two-dimensional solution accounted for 74% of the variance, with a stress value of 0.20 after 13 iterations (see Figure 11b). Again, the level of significance for this stress value is *p* < 0.05 (Klahr, 1969), and this significance level (based on 12 stimuli rather than 14, as in our study) is slightly more stringent than needed. Visual inspection of Figure 11b indicates again the presence of two major clusters of tasks: the first, on the positive side of Dimension 1, included the image generation (within grids and brackets), image maintenance, image rotation, motor imagery, spatial imagery, and image scanning tasks. These tasks are essentially mediated by the dorsal system, and all involve a spatial judgement. Although we had classified the word imagery task as requiring “mixed” processing, because it involved objects

(the words) and spatial judgments, it turned out to be more closely associated with the spatial tasks. The second cluster, on the negative side of Dimension 1, included the face imagery, object imagery, color, auditory, size, and tactile imagery tasks. These two last tasks in the imagery condition were much more associated with the ventral component than in the perception condition. The reliance on the ventral system to retrieve visual information from long-term memory seems to be greater in the imagery condition than in the perception condition.

Again, we interpreted the vertical dimension as the degree of complexity of the stimuli. However, the complexity of the imagery conditions was not the same as that of the perceptual conditions. On the low end of Dimension 2, the image rotation and motor rotation tasks were more complex than the spatial task, probably because participants had to coordinate the visual perceptual input with the image rotation. As in the perception condition, the color task was one of the most complex. On the high end of Dimension 2 were tactile and word imagery, as in the perception condition. However, to our surprise, the imagery condition in the object imagery task did not cluster with the more complex tasks, which we expected because it involved sentence processing. Perhaps, in the imagery condition, the task is less complex because participants do not need to explore the entire visual field to determine which part of the complex visual display is important. In the imagery condition, after hearing the sentence, participants have a cue as to which part of the object to visualize, which thereby reduces the complexity of the necessary representation.

Next, we correlated the values of the tasks along the two dimensions in the two conditions. Dimension 1 in the perception conditions correlated significantly with Dimension 1 in the imagery conditions (r = 0.87, *p* = 0.0001), which indicates that the dorsal/ventral interpretation applied to both conditions. However, we found only a trend for Dimension 2 in the perception conditions to be significantly correlated with Dimension 2 in the imagery conditions (r = 0.46, *p* = 0.09). This result suggests that Dimension 2 may not represent the same variable in the two conditions.

### 3.2 Interpretation of the MDS dimensions by multiple regression analyses

MDS is an exploratory form of data analysis. As such, it inspires possible interpretations but cannot be regarded as definitive. Thus, we next used multiple regression analyses to buttress our interpretations of the MDS dimensions. We determined a number of attributes that in principle could affect performance on the IPB tasks and rated all tasks on a scale from 1 to 7 for each attribute. The author and a collaborator performed these ratings, both being experts in the field of vision. Among these attributes were as follows: the degree of dorsal/ventral processing required, the degree of auditory processing, the number of stimuli, the degree of concentration, memory load, the degree of verbal processing, the number of processes involved, the cerebral hemisphere predominantly involved, visual complexity, verbal complexity, and the degree of stimulus complexity (whether visual or verbal). Whenever these ratings were different for the perceptual and imagery conditions, the appropriate ratings were used in the analysis. These ratings served as dependent variables, and we regressed them against the MDS coordinates separately in the perception and imagery conditions. Tables 3a, b show the multiple correlation and multiple regression coefficients for each MDS dimension as a function of task attributes and their significance levels.

**Table 3A:** Multiple *r* and multiple regression coefficients for predicting task attributes from MDS dimensions in perception.

**Attribute**	**Multiple *r***	**Dimension 1**	**Dimension 2**
Auditory	0.84^**^	−0.75^****^	−0.54^*^
Dorsal/Ventral	0.86^*****^	0.99^******^	−0.02
Verbal processing	0.59	0.19	0.29
Number of stimuli	0.45	−0.22	0.22
Concentration	0.50	−0.32	−0.63
Number of Processes	0.23	0.12	0.22
Hemisphere	0.37	0.02	−0.27
Visual complexity	0.33	0.10	−0.33
Verbal complexity	0.70^*^	−0.60^**^	−0.02
Stimulus complexity	0.83^****^	0.04	−0.61^*****^
Familiarity	0.71^*^	−0.58^*^	0.65^*^

Significance values: ^*^p < 0.05, ^**^p < 0.01, ^***^p < 0.005, ^****^p < 0.001, ^*****^p < 0.0005, and ^******^p < 0.0001.

**Table 3B:** Multiple *r* and multiple regression coefficients for predicting task attributes from MDS dimensions in imagery.

**Attribute**	**Multiple *r***	**Dimension 1**	**Dimension 2**
Auditory	0.67^*^	−0.72^**^	−0.03
Dorsal/ventral	0.86^****^	0.97^*****^	−0.27
Verbal	0.56	0.27	*0*.17
Number of stimuli	0.54	−0.35^*^	−0.07
Concentration	0.32	−0.40	−0.09
Memory load	0.51	0.53	−0.51
Number of processes	0.21	0.14	0.18
Hemisphere	0.45	0.14	−0.29
Visual complexity	0.50	0.96	−0.31
Verbal complexity	0.60	−0.49^*^	−0.20
Stimulus complexity	0.78^****^	−0.30	−0.75^***^
Familiarity	0.70^*^	−0.77^**^	0.29

Significance values: ^*^p < 0.05, ^**^p < 0.01, ^***^p < 0.005, ^****^p < 0.001, ^*****^p < 0.0005, and ^******^p < 0.0001.

Dimension 1 significantly correlated most strongly with the dorsal/ventral ratings in both perception (r = 0.87, *p* = 0.0001) and imagery (r = 0.86, *p* = 0.001), confirming that it reflects the dorsal/ventral continuum. The correlations between this attribute and Dimension 2 were not significant in either perception (r = −0.02, *p* > 0.05) or imagery (r = −0.27, *p* > 0.05). However, Dimension 2 was significantly correlated with the stimulus complexity ratings in the perception condition (r = 0.83, *p* = 0.001) and imagery condition (r = 0.78, *p* = 0.003). Stimulus complexity was not correlated with Dimension 1 in the perception condition (r = 0.04, *p* > 0.05) or imagery condition (r = −0.30, *p* > 0.05).

It is important to note that the dorsal/ventral distinction explained the Dimension 1 much better than all other factors in both perception and imagery. No other attribute than stimulus complexity seemed to account for variation along Dimension 2.

Could the results simply reflect differences in task difficulty? Although the ventral tasks were generally more difficult than the spatial tasks, because the MDS input was the inter-correlation among tasks (rather than the response times *per se*), we can exclude difficulty as an explanation for Dimension 1. The correlations are, in fact, independent of the magnitude of the absolute value of the response times. Furthermore, although the difficulty was uniformly distributed across the entire range, two clear clusters were obtained. If difficulty were at the root of Dimension 1, we would have expected greater homogeneity, with perhaps only one cluster. Instead, we found three very separate clusters in the perception condition and two in the imagery condition.

### 3.3 Factor analyses

We used principal components analysis (PCA) to provide an independent validation of the pattern of results obtained with MDS. The data were rotated using the varimax procedure. Because we posited that all tasks relied on dorsal, ventral, or a mixture of both types of processing, we sought to determine whether factor loadings would reflect these variables. In the perception condition, the first two factors explained 44% and 32% of the variance in the rotated factors in perception and imagery, respectively. In the imagery condition, the first two factors explained 40% and 28% of the variance in the rotated factors, respectively. All eigenvalues were >1, indicating that loadings on the factors considered were significant. Factor loadings > 0.40 were retained for the inclusion of tasks in a given factor.

In the perception condition, Factor 1 had a high loading on spatial perception, image rotation, word, motor imagery, image maintenance, image generation (grids and brackets), and image scanning tasks. Therefore, Factor 1 clearly represented tasks mediated by the dorsal system. Factor 2 showed high loadings on face imagery, object imagery, color imagery, and auditory imagery tasks. Factor 2, then, reflected predominantly tasks mediated by the ventral system. The size imagery and tactile imagery tasks had somewhat moderate loadings on both the ventral and dorsal factors, indicating that they involved, as expected, mixed dorsal/ventral processing (see Table 4a). Interestingly, ventral tasks loaded negatively with the second unrotated factor, while dorsal tasks loaded positively with that factor, indicating that dorsal and ventral tasks are independent, but correlated. The unrotated and rotated factor loadings are shown in Table 4a.

**Table 4A:** Principal components analysis of the perceptual correlation matrix.

**IPB task**	**Unrotated component loadings**		**Rotated component loadings**	
	**1**	**2**	**1**	**2**
Face	0.491	−0.695	0.004	**0.850**
Object	0.519	−0.530	0.152	**0.726**
Spatial	0.354	0.196	**0.404**	0.025
Tactile	0.557	−0.196	0.364	**0.465**
Rotation	0.631	0.124	**0.598**	0.235
Word	0.589	0.609	**0.824**	−0.196
Motor	0.719	0.360	**0.800**	0.084
Color	0.475	−0.655	0.048	**0.808**
Maintenance	0.777	0.257	**0.793**	0.201
Auditory	0.474	−0.573	0.091	**0.738**
Grids	0.851	0.207	**0.829**	0.283
Brackets	0.702	0.151	**0.673**	0.250
Size	0.731	−0.103	**0.561**	**0.480**
Scanning	0.533	0.255	**0.586**	0.072

Bold values correspond to task loadings on rotated factors > 0.40.

In the imagery condition, Factor 1 loaded on dorsal tasks: spatial imagery, image rotation, word imagery, motor imagery, image maintenance, image generation (grids and brackets), and image scanning tasks. Factor 2 loaded on ventral tasks, including face, object, tactile, color, auditory, and size imagery tasks. In the imagery condition, the tactile and size imagery tasks were more clearly associated with the ventral factor, perhaps because there is a greater reliance on long-term memory retrieval of the objects than in the perception condition. The unrotated and rotated factor loadings are shown in Table 4b.

**Table 4B:** Principal components analysis of the imagery correlation matrix.

**IPB task**	**Unrotated component loadings**		**Rotated component loadings**	
	**1**	**2**	**1**	**2**
Face	0.359	−0.599	0.005	**0.698**
Object	0.501	−0.342	0.257	**0.549**
Spatial	0.666	0.250	**0.701**	0.124
Tactile	0.285	−0.388	0.052	**0.481**
Rotation	0.606	0.147	**0.597**	0.182
Word	0.541	0.110	**0.522**	0.181
Motor	0.563	0.109	**0.541**	0.193
Color	0.168	−0.393	−0.055	**0.423**
Maintenance	0.635	0.362	**0.731**	0.012
Auditory	0.610	−0.652	0.194	**0.872**
Grids	0.617	0.495	**0.783**	−0.112
Brackets	0.780	0.269	**0.808**	0.166
Size	0.715	−0.545	0.338	**0.833**
Scanning	0.648	0.356	**0.739**	0.024

Bold values correspond to task loadings on rotated factors > 0.40.

The dorsal/ventral distinction, then, accounted well for both the perception and imagery data. These results dovetail nicely with the results from the MDS analysis, even though the percentage of total variance accounted by the factors was relatively low. In summary, we are confident that the first two factors reflect dorsal/ventral processing in both perception and imagery.

## 4 Discussion

The IPB is the first battery to assess a wide range of comparable perceptual and imagery processes. The results of multivariate statistical analyses (MDS and PCA) converged in demonstrating that two major clusters or factors underly the performance of young healthy participants on the IPB. The results suggest that the IPB tasks assess aspects of processing that rely on the dorsal and ventral systems. Moreover, the results are consistent with the idea that imagery and perception draw on common mechanisms within the two processing streams. However, perception and imagery also differed in some respects. For example, we interpreted Dimension 2 of the MDS solution as reflecting the degree of stimulus complexity. However, stimulus complexity was not equivalent in perceptual and imagery conditions, and probably nor were memory load, concentration, and other factors. More processes may have been involved in the imagery conditions than in the perception conditions, but we can only speculate about how various processes may have given rise to this dimension.

This study revealed the segregation of the dorsal and ventral systems at a purely behavioral level, in both imagery and perception. Previous studies have demonstrated this segregation in young participants using brain imaging techniques, such as Positron Emission Tomography (PET) and functional Magnetic Resonance Imaging (fMRI) (e.g., Haxby et al., 1991). Similarly, a study by Chen et al. (2000) examined performance in a set of visuospatial perceptual tasks and found clusters that represented dorsal vs. ventral processing. However, the tasks of Chen et al. (2000) relied predominantly on dorsal stream processing. Several of our tasks predominantly relied on ventral processing. Moreover, our study allowed us to extend the results to mental imagery.

However, we must sound a note of caution regarding the interpretation of the first MDS dimension. Even though the distinction between spatial vs. object processing characterizes the results, other distinctions have been associated with these two systems. Goodale and Milner (1992), Whitwell et al. (2015), and Ganel and Goodale (2019) claimed that whereas the ventral system is associated with the perception of object characteristics, the dorsal system may be involved in action. Although the interpretation of the functions of the two systems may not be entirely clear, there is strong agreement that they carry out different classes of processes. For present purposes, the important result is the similarity between imagery and perception, providing additional evidence of underlying shared mechanisms.

We also note that although the behavioral clustering is consistent with this interpretation, future studies that conduct neuroimaging, while participants take the IPB, are ultimately required to fully validate the neural basis of these tasks.

In addition, one could try to characterize the results in less interesting terms. For example, the spatial tests often did not involve auditory input, whereas most of the ventral tasks did involve auditory input—particularly in the imagery conditions. Therefore, one could argue that the configurations are due to the auditory/nonauditory nature of the tasks. This characterization fails to explain why the spatial task, which had auditory input, clustered with the other spatial tasks; if the auditory/non-auditory nature of the input consistently influenced the results, we would have expected the spatial task to cluster with the ventral tasks. The regression analyses also indicated that the dorsal/ventral distinction provided the best fit to the data, explaining a greater portion of variance than other factors, such as the amount of auditory processing or the verbal complexity of the stimuli.

One also might try to argue that task difficulty underlies the observed differences, given that the ventral tasks generally were more difficult (participants made more errors and were slower). However, this distinction cannot explain the results because the multivariate statistics were carried out on the correlations between response times in the different tasks. They were, therefore, independent of the magnitude of the relative difficulty levels of the tasks.

A major limitation of this study is that we typically did not have a direct or objective way to confirm that participants actually engaged in mental imagery in the imagery tasks. We have three responses to this concern. First, alternative cognitive strategies (e.g., verbal reasoning, implicit rule-based judgments, or counting) would generally require more time and not exhibit characteristic response time patterns found when imagery is used. For example, one could argue that participants counted the number of segments in the letters used in the image generation tasks, and that counting produced the longer response times for probes on segments further in the sequence. But counting would have produced larger differences than the millisecond effects we found. Second, in some tasks, such as mental rotation, participants could have used a “spatial reasoning strategy” (cf. Kay et al., 2024)—but such a strategy itself would presumably have drawn on the dorsal system and could draw on spatial imagery. Third, it is possible that in some cases, participants may have responded by drawing on semantic memory. In order to do so, however, they would need to have stored the information explicitly. Previous work on size comparisons by Kosslyn et al. (1977) provided evidence that images are used when to-be-compared objects fall into the same overlearned size category; in contrast, when objects are in different categories, the participants apparently used the category labels themselves to perform size comparisons—without using imagery. These findings suggest that when items are similar enough to be in the same general category, imagery is likely to be used. An exception, however, would occur when the comparison had been made explicitly before, in which case the judgment itself could be stored. We thus used comparisons that were unlikely to have been made previously. The same logic applies when we asked about specific attributes of objects, using items similar to those previously validated by Eddy and Glass (1981): The attributes we probed were unlikely to have been considered previously.

In any case, in many tasks, we selected items based on previous ratings of how often participants used imagery. Behavioral and functional brain imaging studies also have provided good evidence that similar tasks engage mental imagery (e.g., see Kosslyn, 1980, 1994).

Another potential issue is that we only used two orders of the tasks. This counterbalancing may not totally account for the effects of practice or fatigue. However, we distributed each type of task (spatial, ventral, and mixed) throughout the sequence, which would minimize a possible confounding between the order and type of task.

Another possible problem is that only two people produced the ratings used in the regression analyses. Ideally, a larger group of raters blind to the hypothesis should have made these ratings—but this was not possible because we needed experts in the field of vision. The two raters made these ratings independently and did not compare notes or consult each other during this process. These precautions reduced at least some potential biases in the multivariate analyses.

Another limitation was the small sample size, which was limited owing to the considerable amount of time required for administration by the IPB.

A main strength of the present study is the development of the IPB itself. This battery includes a large number of tasks that assess different and comparable aspects of visual perception and mental imagery, which appear to be mediated largely by the dorsal or ventral visual pathways. We also showed that several other factors, such as memory load and concentration, could not explain the results. This test battery can be used in many contexts, which can serve both to develop it and validate it further.

In conclusion, we have described a new test battery that assesses a wide range of perceptual and mental imagery processes. We were able to show that these tasks rely on processing from the dorsal and ventral systems, and using multivariate statistics, we were able to show this distinction at the behavioral level in young healthy participants.

## Data Availability

The raw data supporting the conclusions of this article will be made available by the authors, without undue reservation.
